# Surface-Vinylated Cellulose Nanocrystals as Cross-Linkers for Hydrogel Composites

**DOI:** 10.1021/acs.biomac.4c01619

**Published:** 2025-03-11

**Authors:** Marcel Kröger, Timo Pääkkönen, Lukas Fliri, Anna F. Lehrhofer, Irina Sulaeva, Antje Potthast, Eero Kontturi

**Affiliations:** † Department of Bioproducts and Biosystems, Aalto University, Aalto FI-00076, Finland; ‡ Nordic Bioproducts Group Oy, Tietotie 1, Espoo 02150, Finland; § Institute of Chemistry of Renewable Resources, Department of Chemistry, University of Natural Resources and Life Sciences, (BOKU), Muthgasse 18, Vienna A-1190, Austria; ∥ Core Facility “Analysis of Lignocellulosics” (Alice), University of Natural Resources and Life Sciences, (BOKU), Konrad-Lorenz Strasse 24, Tulln, Vienna A-3430, Austria

## Abstract

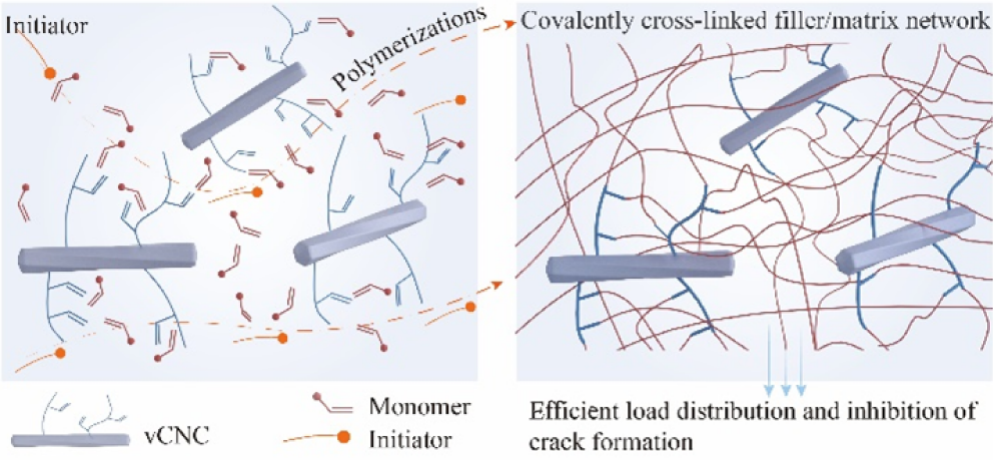

Cellulose nanocrystal (CNC) fillers have been shown to significantly improve the performance of polymer composites and hydrogels, elevating both strength and toughness. Polymer grafting from the surface of the nanocrystals has been employed to enhance matrix–filler interactions and keep the fillers dispersed within the matrix. However, such approaches often rely on multistep syntheses and diligent process control. Here, we propose modifying the nanocrystal surface to carry vinyl moieties, turning the particles into cross-linking comonomers. Using allyl glycidyl ether in an aqueous modification route, we were able to decorate the CNCs with varying amounts of vinyl moieties. Subsequent dispersion in 2-hydroxy methacrylate and thermally initiated free radical polymerization yielded composite materials that showed superior mechanical performance compared to those obtained from monomeric cross-linkers and unmodified CNCs. The large discrepancies in the observed glass transition temperatures of the obtained materials suggest, however, that the impact of the fillers on the polymerization kinetics is significant and less easily explained.

## Introduction

The concept of improving the material properties of polymers by including filler particles has been exploited since the 1960s. It has since been shown that the synergistic effects are heavily dependent on the size and shape of the filler particles much more than their intrinsic properties.^1−3^ This is due to matrix–filler interactions along the interface as well as filler–filler interactions.^4,5^ Given their availability and distinct one-dimensional geometry originating from the fibrous nature of cellulose, cellulose nanomaterials are of particular interest in this context.^3,6,7^ Cellulose nanocrystals (CNCs), in particular, have been utilized as composite fillers for three decades already.^8^ CNCs are rod-like particles with aspect ratios in the range of 10–100, and they are isolated from cellulose fibers by selective hydrolysis of noncrystalline domains.^9^ The more crystalline segments are defined through their lower surface energy compared to the noncrystalline regions, which increases their resistance to the acid treatment but also to desired chemical modifications due to steric hindrance.^10,11^ Instead, given the abundance of hydrogen bond donor and acceptor moieties on the CNC surface, their tendency for aggregation is notorious, and multiple methods for preventing the aggregation have been proposed,^12^ among which the most prominent one is polymer adsorption or grafting on the CNC surface.^13,14^

The grafting strategies include both “grafting from” the cellulose surface, prominently by means of immobilized atom transfer radical polymerization (ATRP) and reversible addition–fragmentation chain-transfer (RAFT) initiators and subsequent polymerizations^15^ and the “grafting onto”-approach of attaching presynthesized or growing polymer chains onto the nanocellulose fillers.^16,17^ As nanocellulose derivatives are considered biocompatible,^18^ multiple uses in biocomposite materials and biomedical applications have emerged.^19,20^ Therefore, nanocellulose fillers are suitable for poly(2-hydroxyethyl methacrylate) (pHEMA), a polymer that finds diverse applications in drug delivery, tissue engineering, ophthalmic products, and dental adhesives.^21,22^ With an emphasis set on the controlled curing of adhesives in particular and similarly scaffolds produced by additive manufacturing, however, grafting-from approaches to obtain nanocellulose composites are less suitable.^12,23^ Additionally, reshaping the composites postsynthesis is laborious and thus less desired.^12,24^

Instead, we present here a detailed survey on poly(hydroxyethyl methacrylate) grafting *through* vinylated CNCs, complete with a demonstration of their performance as composite fillers. This requires a vinylation step of the cellulose surface^25^ followed by free radical polymerization in hydroxyethyl methacrylate, which potentially yields covalent polymer-nanofiller networks. Our proposal is to use vinylated CNCs as cross-linkers for pHEMA hydrogels, thus combining their role as reinforcing filler particles and cross-linking agents. Although vinylation has been presented for nanocellulose in a small number of accounts, there is only one report on utilizing it for grafting through polymerization.^26^ However, that approach utilized undecenoyl chloride to introduce the double bond, which is unsuitable for hydrophilic monomers like HEMA and certainly would not work for hydrogel composites, such as the ones introduced in this study. Grafting through polymerization with vinylated CNCs offers the unique advantage of enabling the said usage of reinforcing CNCs as cross-linkers in the covalent cross-linking network of pHEMA.

## Materials and Methods

### Materials

(2,2,6,6-Tetramethylpiperidin-1-yl)oxyl (TEMPO)-oxidized CNCs were prepared from bacterial cellulose provided by our industrial collaborators and from bleached hardwood pulp (birch) as described previously^27,28^ by HCl gas hydrolysis and subsequent TEMPO-oxidation. Bacterial cellulose-derived CNCs were dispersed by microfluidization, wood CNCs were dispersed using an ATREX G-series processor (Megatrex OY, Lempäälä, Finland). Milli-Q ultrapure water, sodium hydroxide (>97%, CAS 1310–73–2, VWR), allyl glycidyl ether (AGE, > 99%, CAS 106-92-3, Sigma-Aldrich), 2-hydroxyethyl methacrylate (HEMA, 97%, CAS 868-77-9, stabilized with 250 ppm MEHQ, Sigma-Aldrich), triethylene glycol dimethacrylate (TEGDMA, 95%, CAS 109-16-0, stabilized with 80–120 ppm MEHQ, Sigma-Aldrich), lithium chloride (LiCl, ≥99%, CAS 7447-41-8, VWR), diammonium persulfate (APS, 98%, CAS 7727-54-0, Sigma-Aldrich), hydrochloric acid solution (HCl, 0.1 M, CAS 7647-01-0, Titripur Reag., Merck), and sodium hydroxide solution (NaOH, 1 M, CAS 1310-73-2, Titripur Reag., Merck) were used without further purification. Dialysis membranes (Spectrum Spectra/Por 1RC, diameter 32 mm, MWCO 6–8 kDa, Fisher Scientific) were wetted in deionized water for 20 min and flushed with deionized water before filling. *N,N*-Dimethylacetamide (DMAc, CAS 127-19-5, Sigma-Aldrich) was purified in-house by distillation before use (≥99.98%, GC-FID). The [P_4444_][OAc]:DMSO-*d*_*6*_ (1:4 wt %) electrolyte used for solution state NMR characterization of the cellulosic materials was prepared according to a previously reported protocol.^29^

### CNC Surface Modification

The reaction conditions were initially optimized for the modification of bacterial cellulose CNCs (for details, see Supporting Information) and then adapted to the wood-based CNCs.

The concentration of the dispersed CNC was increased from 1% to 10% by the rotary evaporation of water. Sodium hydroxide pellets (60 wt % with regard to the cellulose, corresponding to 6 wt % overall) were ground in a mortar and mixed with the obtained CNC gel. Upon dissolution of the sodium hydroxide, the gel reverted to a viscous fluid, and a slight yellow discoloration was observed. To this viscous dispersion, 10–40 wt % of allyl glycidyl ether were added. The solubility limit of allyl glycidyl ether in water is 14%, and accordingly, phase separation was observed for large amounts of allyl glycidyl ether. Homogeneous mixtures were obtained after vigorous magnetic stirring; in the case of small scales and high amounts of allyl glycidyl ether, the mixing was aided by vortex stirring using an IKA Vortex stirrer.

The modification reaction was conducted at room temperature for 72 h, after which the reaction was ended by adding 1 M HCl solution until a pH of 3 was reached. The parameters for the two modification products considered here are shown in Table 1.

**Table 1:** Reactants Used in the Modification Reaction of CNCs with Low and High Amounts of AGE

	lDS-vCNC	hDS-vCNC
Dry mass of suspended CNC (1 wt % in water)	4.01 g	10.05 g
Concentration after water removal	10 wt %	9.2 wt %
NaOH (resulting concentration)	6.1 wt %	6.1 wt %
Mass of AGE (wt %)	2.68 g AGE (6.7 wt %)	37.7 g AGE (34.28 wt %)
Reaction time	72 h	72 h
Recovered product (dry mass)	4.10 g	9.97 g

### Purification of Modified CNC

The reaction mixture previously set to pH 3 was centrifuged for 30 min at 10000 *g* to separate the CNC from the supernatant solution containing the side products. The precipitate was redispersed in deionized water and sonicated for 10 min. The resulting dispersion was dialyzed for 7 days against Milli-Q water. The dialysis tubes were filled half full, given the significant influx of water in the initial stage of the dialysis process. After dialysis, the volume of the obtained product was adjusted to 1 L, and the particles were redispersed by bath sonication.

### Polymerization

The concentration of the purified and redispersed modified CNCs was adjusted by careful rotary evaporation. This way, CNC dispersions in water with up to 5 wt % CNC were produced. Under stirring, 1 wt % of ammonium persulfate (APS) initiator was dissolved in these suspensions, before the weight of this obtained mixture was doubled by adding monomer (weight ratio CNC suspension:monomer 1:1).

Alternatively, for comparison, aqueous solutions containing 1–5 wt % of TEGMA were used to dissolve 1 wt % of APS initiator and enough HEMA to obtain a 1:1 wt/wt mixture of H_2_O/TEGDMA/APS:HEMA. Subsequently, a precipitation polymerization was carried out by heating the aqueous solution of the monomer and the initiator with dispersed CNC particles to 70 °C for 3 h. At this point, an opaque polymer dispersed in water was obtained. At full yield, the obtained material is composed of 50 wt % polymer composite and 50 wt % water. Ideally, both the initiator and the filler particles are transferred quantitatively into the polymer, yielding a pHEMA composite containing 1 wt % initiator and up to 5 wt % CNC filler, leaving behind pure water. As reshaping the obtained polymer proved challenging, the polymerizations were carried out in Teflon molds, giving access to 40 mm × 10 mm × 0.4 mm specimens (see Supporting Information for the exact dimensions of the molds). To inhibit the evaporation of both water and the monomer, the surface of the mold was covered with a polyethylene film, allowing for bubble-free sealing of the compartments. Initially, surface adhesion keeps this film in place; then the curing polymer acts as an adhesive. Over the course of the polymerization, the cover adapts to volume expansion due to the initial temperature increase and volume contraction over the course of the polymerization.

In a typical experiment, 0.1 g of APS was dissolved in 10 g of CNC dispersion of preadjusted concentration. After complete dissolution, 10 g of HEMA was added to yield the monomer formulation, which was subsequently cured to produce at least 18 individual polymer specimens.

### Analytics

#### Fourier-Transform Infrared Spectroscopy (FTIR)

ATR-FTIR spectra were recorded on a PerkinElmer Spectrum Two FTIR instrument in the range of 400–4000 cm^–1^. The displayed spectra were baseline-corrected using the Spectrum 10.6.2 software provided by PerkinElmer, and the total area under the curves was normalized with OriginPro 2023 software. In order to achieve matching intensities for the signals at 3000–3600 cm^–1^, highlighting changes in the other areas of the spectra, the spectra for lDS- and hDS-vCNCs were scaled up to a total area of 1.64, with the spectrum of unmodified CNCs normalized to a total area of 1.

#### Raman Spectroscopy

Raman spectra were obtained using a Renishaw inVia Confocal Raman microscope operated with WiRE 5.3 software. The freeze-dried cellulose samples were compressed by hand into small discs. These were analyzed under 100-fold magnification, allowing for the analysis of an area similar in size to that considered in ATR-FTIR. The spectra were recorded in the range between 400 cm^–1^ and 3200 cm^–1^. The spectra were normalized as described for the FTIR data.

#### Nuclear Magnetic Resonance Spectroscopy (NMR)

Solution state NMR spectra were recorded on a Bruker NMR AVANCE NEO 400 spectrometer. The cellulosic materials were dissolved in [P_4444_][OAc]:DMSO-*d*_6_ (1:4 wt %) electrolyte (“NMR electrolyte”) at a concentration of 5 wt % at 65 °C for 16–24 h, closely following a previously reported protocol.^29^ To avoid solubility issues in the electrolyte system, the TEMPO-oxidized CNCs were dissolved in the protic form.^30^ All spectra were recorded at a measuring temperature of 65 °C. The cellulosic samples were routinely characterized by quantitative ^1^H, diffusion-edited ^1^H, and multiplicity-edited ^1^H–^13^C HSQC experiments. Attempts to directly confirm the covalent attachment of the allyl glycidyl ether groups on the cellulose surface by ^1^H–^13^C HMBC experiments were not successful. For indirect confirmation, the hDS-vCNC sample was stirred in DMSO-*d*_6_ (1 g) at 65 °C for 16 h, and the supernatant liquid was analyzed for solubilized polymeric fractions.

The quantitative ^1^H spectra were evaluated to roughly estimate the amount of introduced vinyl groups after aggressive baseline correction and peak fitting using the software *Fityk*.^31^ The peak areas of the C1–H area of the glycopyranose units (I_C1–H_ ∼ 4.2–4.5 ppm) and the CH_2_ group adjacent to the vinyl moiety of the allyl glyceryl ether (I_CH2_ ∼ 3.8–4.1 ppm) were used to calculate the moles of substitution according to the equation:
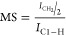


#### Gel Permeation Chromatography (GPC)

The samples were treated according to the standard protocol for FDAM-cellulose labeling (7 days in a shaking water bath at 40 °C).^32^ The freeze-dried sample (approximately 12 mg) was washed on a Büchner funnel with filter paper (Lab Logistics Group GmbH, Meckenheim, Germany) by vacuum filtration with 0.1 M HCl (2 × 5 mL), ethanol (2 × 5 mL, 96%), and DMAc (2 × 3 mL) and put in a 4 mL glass vial. To the DMAc-wet cellulose sample, 0.5 mL of FDAM labeling solution (approximately 0.125 mol/L in DMAc) and 1.5 mL of DMAc were added. The vial was closed and shaken at 40 °C for 7 days. After filtration and washing with DMAc (3 × 4 mL), the samples were dissolved in DMAc/LiCl 9% (w/v) (c ∼ 10 mg sample/mL) on the shaker at RT (24 h), diluted with pure DMAc (approximately 1:1), and filtered through a 0.45 μm syringe filter before injection.

Analysis was performed according to published procedures using a size exclusion/multidetector (GPC-MALLS-RI) system. The system was composed of a Bio-Inert 1260 Infinity II HPLC pump, an HP Series 1100 autosampler (G1367B; Agilent Technologies, Waldbronn, Germany), a MALLS detector (Wyatt Dawn DSP with a diode laser, λ = 488 nm, Wyatt Inc.), a refractive index detector (Shodex RI 71), and a fluorescence detector (Shimadzu RF 535, Shimadzu GmbH, Korneuburg, Austria; excitation, 252 nm; emission, 323 nm). Four SEC columns (Styragel HMW 6E Mixed Bed, 15–20 μm, 7.8 mm i.d., 300 mm length, Waters GmbH, Vienna, Austria) with one Agilent GPC guard column (PL gel, 20 μm, 7.8 mm i.d., 50 mm length; Agilent Technologies, Waldbronn, Germany) were used as a stationary phase. As a mobile phase, *N,N*-dimethylacetamide/lithium chloride (0.9%, w/v; filtered through a 0.02 μm filter) was used and operated at a flow rate of 1 mL/min. For each measurement, samples were filtered through a 0.45 μm syringe filter, and a sample volume of 100 μL was injected with a 45 min run time. The system was operated at room temperature. The molecular weight distribution and the statistical moments were calculated based on a refractive index increment of 0.140 mL/g for cellulose in *N,N*-dimethylacetamide/lithium chloride (0.9% w/v) at 488 nm. The raw data was processed with Astra 4.73 (Wyatt Technologies), GRAMS/AI 7.0 (Thermo Fisher Scientific), and OriginPro 2023 software.

#### Conductometric Titrations

Conductometric titrations were carried out on the dialyzed fibers following a slightly adapted protocol to the one described by Foster et al.^33^ An aliquot of the dispersed, dialyzed CNCs containing 300 mg of dry fibers was added to 500 mL of degassed Milli-Q water. Initial protonation of the carboxylate groups was ensured by adding 8 mL of 0.1 M HCl solution. After determining the initial weight, the samples were titrated with 15 mL of 0.1 M sodium hydroxide solution, dosed with a Metrohm 651 Dosimat at 0.1 mL/min. Conductivity curves were recorded by using a Metrohm 712 conductometer operated with Tiamo 1.2.1 software.

For the charge determination, the curves were normalized by multiplying with the volume (determined from the initial sample weight assuming a density of 1 kg/m^3^ and an added volume of NaOH). Subsequently, linear fitting was carried out according to SCAN-CM 65-02-1, using OriginPro 2023 software. The COOH content of the starting CNCs was 1150 μmol/g, amounting to a degree of substitution of 0.19 with respect to all anhydroglucose units in a CNC (see Table 2).

**Table 2:** Calculated Statistical Moments Obtained by MALLS and Carboxyl Content Obtained by FDAM Labelling

Sample	Calculated injected mass [mg]	*M*_n_ (kDa)	*M*_w_ (kDa)	M_*z*_ (kDa)	Đ	[COOH] (μmol g^–1^)	[COOH] content from titration (μmol g^–1^)
Unmodified CNC	0.175	17.03	29.47	47.91	1.73	516	1150
lDS-vCNC	0.159	18.8	32.74	55.86	1.74	289	610
hDS-vCNC	0.086	21.06	47.07	132.1	2.23	315	480

#### Atomic Force Microscopy (AFM)

Silicon substrates were immersed in a 3.5 wt % PEI solution (*M*_w_ = 2000–4000 g/mol) for 15 min, rinsed carefully with deionized water, and air-dried. Then, 50 μL of 0.01 wt % pCNC dispersion was spin-coated at 4000 rpm. The substrates were imaged using a Bruker Multimode 8 AFM in tapping mode. Cantilevers of the model NCHV-A by Bruker with force constants of 42 N/m and 320 kHz resonance frequency were used. The obtained images were treated with plane-fitting and flattening to achieve a smooth baseline, before height and length distributions were collected using the NanoScope Analysis 3.00 software.

#### Wide-Angle X-ray Scattering (WAXS)

Wide-angle X-ray scattering data were obtained by using a Xenocs Xeuss 3.0 SAXS/WAXS system (Xenocs SAS, Grenoble, France). The system consists of a microfocus X-ray source (sealed tube) with a Cu target and a multilayer mirror, which yields a parallel beam with a nominal wavelength of 1.542 Å (combined Cu K-α_1_ and Cu K-α_2_ characteristic radiation). The source operates at 50 kV and 0.6 mA. The beam is collimated by a set of variable slits, and the experiments were conducted with a beam size of 0.7 mm. As the system does not include a beam stop, direct measurements of sample transmission were conducted. The data were acquired by using an area detector (Eiger2 R 1M, Dectris AG, Switzerland). The sample-to-detector distance was calibrated by measuring the diffraction from a known LaB_6_ standard sample.

Freeze-dried cellulose samples were analyzed by sealing the analyte in aluminum washers using Kapton films. Scattering contributions from the empty chamber and the two layers of Kapton film were determined by measuring an empty washer under the same conditions, and the obtained intensities were subsequently subtracted from the azimuthally averaged data of the samples.

#### Mechanical Testing

Stress–strain measurements were conducted using a TA.XT plus texture analyzer by Stable Micro Systems, equipped with miniature tensile grips (15 mm × 5 mm). The polymer samples were stretched up to 250% elongation at a rate of 1 mm/s. The slightly concave shape of the top surface of our specimen was considered using eq S2. Thus, calculated cross-sectional areas were used to normalize the measured stresses.

#### Differential Scanning Calorimetry DSC

DSC measurements were performed on a DSC 3+ instrument (Mettler Toledo). The obtained polymer hydrogels were frozen in liquid nitrogen, crushed in a mortar, and freeze-dried. In a typical experiment, 20 mg of the dry polymer was then heated from 25 to 180 °C at 10 K/min in 40 μL aluminum crucibles. Measurements were performed in triplicate, although the first measurements of each set, regularly showing traces of lingering moisture being evaporated, were not considered.

## Results and Discussion

### CNC Modification and Characterization

The parameters used in the synthesis of the two batches of particles considered here are shown in Table 1. The CNCs used here were derived from wood. For comparative experiments performed on TEMPO-oxidized bacterial nanocellulose, see Supporting Information.

The chosen conditions for the reaction with allyl glycidyl ether were adapted from a previously reported protocol for a homogeneous modification of cellulose dissolved in a NaOH/urea solvent system.^25^ By conducting the reaction in a heterogeneous approach in the absence of urea, it was attempted to restrict the modification to the surface of the CNCs. We increased the cellulose concentration to 10 wt % to limit the amount of water in the system. The CNCs were forming a gel, which reverted to a viscous fluid when sodium hydroxide was added. The increased concentration theoretically increases the reaction speed, but crucially, the rather low solubility of allyl glycidyl ether in water of 14 wt % is reached when 2 equiv of allyl glycidyl ether per glucopyranose unit are added to the 10 wt % dispersion of the CNC in water. Adding more allyl glycidyl ether causes the formation of an emulsion, which can be stabilized by CNCs in the system. The presence of reactive cellulose on the interphase of an allyl glycidyl ether droplet should then promote the grafting reactions via exclusion of water. With increasing consumption of the epoxy moiety, however, the system reverts to a homogeneous cellulose dispersion in an aqueous solution of the reactant.

**Figure 1:**
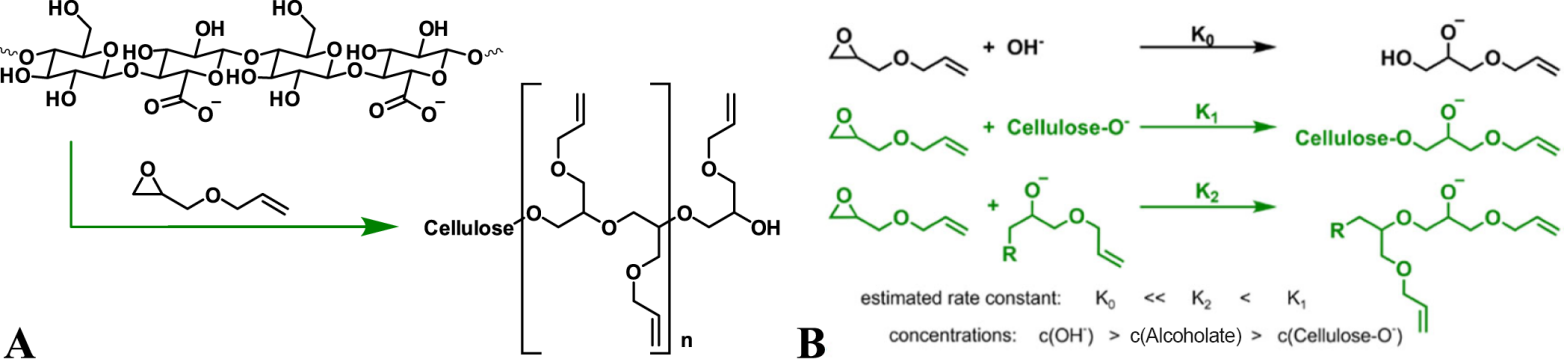
Modification reaction (A) and comparison to possible side reactions (B). The relative rate constants is based on Tollini et al.^35.^

While proven to be effective here and in the literature,^34^ the modification strategy for our CNCs suffers from poor selectivity due to the abundance of nucleophiles in the system. The intended modification reaction is shown in Figure 1A, while Figure 1B compares possible competing reactions.

The addition of epoxy units through nucleophilic ring-opening depends on the concentration of nucleophiles and their nucleophilicity.^35^ Apart from the desired alcoholates derived from cellulose and successfully added allyl glyceryl units, our system contains a large amount of hydroxyl ions. Despite being far less nucleophilic than alcoholates, the reaction between hydroxyl ions and AGE imposes significant limits on the achievable yield in the modification reactions (see Figure 1B).

Obviously, these limitations could be avoided by quantitatively removing the water from the system, using a strong, non-nucleophilic base, and conducting the reaction in an aprotic solvent. In the absence of hydroxyl ions, no allyl glyceryl ether can be formed as a side product, leaving only the desired reaction path. Practically, however, the quantitative removal of all water from the system, while keeping the nanocrystals dispersed in the reaction medium, is nearly impossible. Additionally, traces of water will be deprotonated quantitatively in the presence of strong bases and therefore still initiate side reactions. Given these challenges, we chose to embrace the side reactions in an aqueous dispersion rather than conducting laborious drying and solvent exchange steps.

A further benefit of the aqueous reaction lies in the abundance of water, ensuring that the reactive hydroxyl groups are likely to be protonated between separate addition steps of epoxy units. This means that the growth of longer oligomers or the full-scale ring-opening polymerization of allyl glycidyl ether is suppressed.

Additionally, the side products formed during the modification process can potentially be hydrolyzed to glycerin and allyl alcohol, while allyl glyceryl ether, specifically, can be reverted to allyl glycidyl ether in a two-step synthesis.^36^

**Figure 2:**
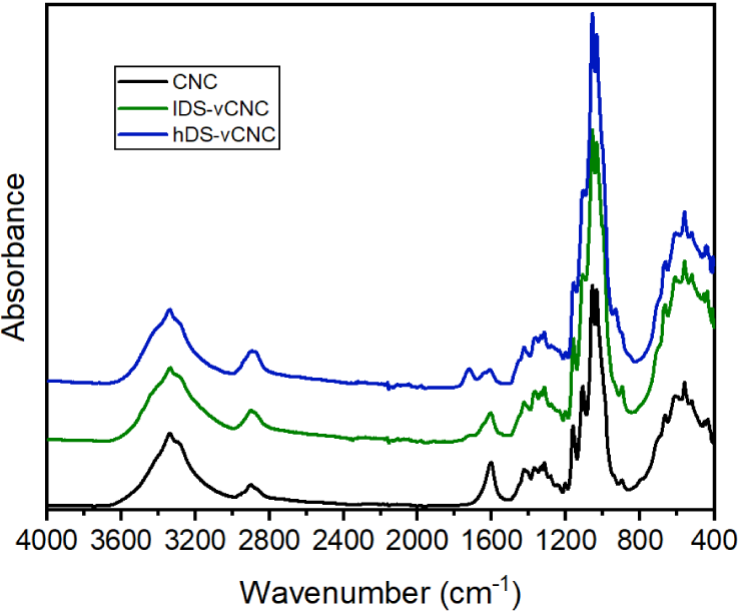
FTIR spectra of CNC before and after modification with low and high amounts of AGE.

**Figure 3:**
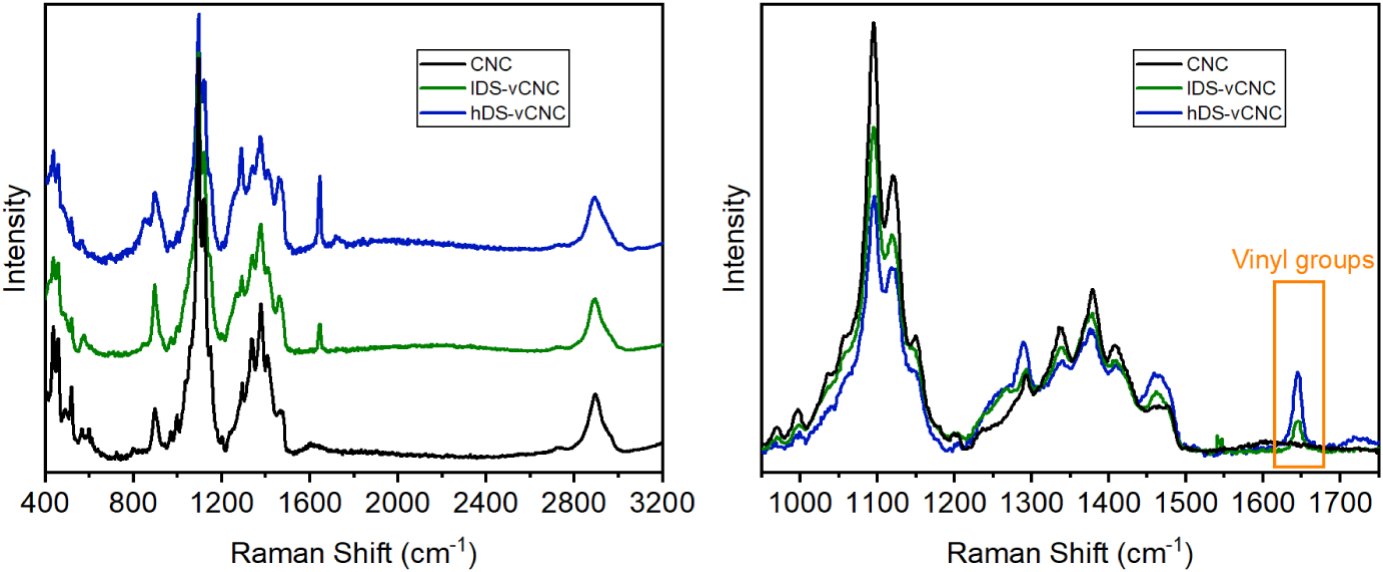
Raman spectra of the CNC before and after modification with low and high amounts of AGE. The right part shows an overlay of the zoomed-in section between 950 and 1750 cm^–1^, showing the emergence of a vinyl band at 1650 cm^–1^.

**Figure 4:**
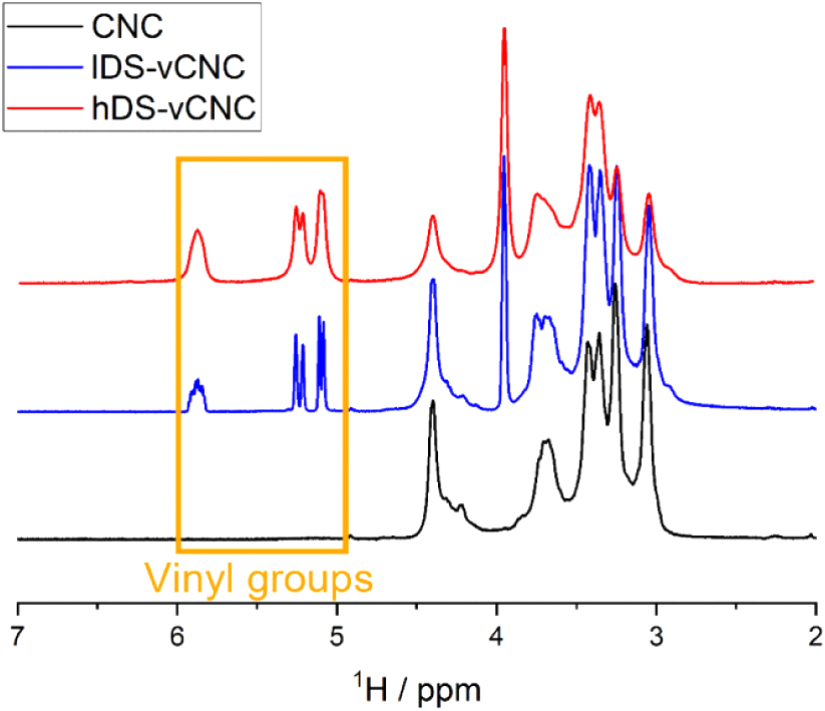
Comparison of the diffusion-edited ^1^H NMR spectra ([P_4444_][OAc]:DMSO-*d*_*6*_ (1:4); 400 MHz; 65 °C) of the CNC before (bottom) and after modification with low (middle) and high (top) amounts of AGE.

After the modification reaction and ensuing dialysis, the obtained vinylated cellulose nanocrystals were thoroughly analyzed by FTIR, Raman, and NMR spectroscopy (Figures 2**–**4).

Consistent with the concept of surface modification, the FTIR and Raman spectra of the modified particles in Figures 2 and 3, respectively, are very similar to the starting material. This is to be expected. While the surface of the particles is exposed for potential modification, the bulk of the particles remains unchanged. Furthermore, when attached, the modifying agent has the same functionalities as the cellulose: ether moieties, an alcohol function as an end group, as well as methylene and methine functionalities. In principle, the FTIR spectra in Figure 2 show two key differences. With an increasing amount of AGE used in the modification reactions, the bands assigned to C–O-stretching vibrations of ethers and alcohols between 900 and 1200 cm^–1^ grow in intensity compared to the signals for O–H- and C–H vibrations above 2800 cm^–1^. This is consistent with the introduction of more ether bonds on the surface of the material, leading to an increase in the mass concentration reflected in the FTIR signal intensity. Additionally, the band appearing at 1600–1650 cm^–1^ related to the carboxylate functionalities of the TEMPO-oxidized starting material, which are deprotonated prior to modification, splits into two smaller bands after modification. The appearance of the second band at 1720 cm^–1^ is most likely due to the protonation of the carboxylate groups. As the dialysis is started in acidic conditions, the carboxylate moieties are largely protonated, and counterions are washed out. Therefore, protonated carboxylates are seen in the FTIR spectra at slightly larger wavenumbers. Additionally, the signal at 1720 cm^–1^ instead of 1740 cm^–1^ has previously been connected to hydrogen bonds,^37^ which can be expected between protonated carboxylates and ether groups in the introduced substituents. The remaining signal at 1600–1650 cm^–1^ can be attributed to residual carboxylate and, to a degree,^38^ adsorbed water.^39^ Any significant signal connected to vinyl groups remains absent in the FTIR spectra. This seems contradictory to published works on detecting and estimating vinyl group contents introduced via silanization by means of FTIR.^40−42^ Considering that the vinyl groups do not carry any dipole moment, it seems reasonable that their signal in FTIR should be marginal. However, in the comparison of the Raman spectra in Figure 3, which detect vibrations of functional groups with changing polarization, it becomes evident that vinyl groups are, indeed, present. This is indicated by a strong band at 1645–1650 cm^–1^, which is unreciprocated in the corresponding FTIR spectra. As such, their invisibility in IR must be due to the limited response of the functional groups and the corresponding bands overlapping with the signal of the deprotonated carboxylic groups and adsorbed water.

To obtain further insights into the occurring transformations, the starting material, the lDS-vCNC and hDS-vCNC samples were investigated by solution-state NMR.^29^ In the unmodified TEMPO-oxidized CNCs, primarily, resonances of the cellulose backbone were visible. In the ^1^H–^13^C HSQC spectra, additionally minor resonances for residual hemicelluloses and oxidized moieties were observed. Compared to previously reported studies on TEMPO-oxidized low DP cellulose model compounds,^30^ no full spin systems of anhydroglucuronic acid groups could be distinguished. This might be due to a lower degree of oxidation and a higher molecular weight of the investigated CNCs. Additionally, a minor peak of formate was observed in the ^1^H spectrum at 8.60 ppm. This peak is associated with β-elimination reactions of C2/C3 carbonyl functionalities in the alkaline electrolyte system, which can be introduced as a side reaction of TEMPO oxidation.^43−45^ This degradative side reaction can also serve as a source for partial depolymerization during modification in an alkaline reaction medium.

After modification, clear signs for allyl moieties were visible in the diffusion-edited ^1^H (Figure 4) and ^1^H–^13^C HSQC spectra of the lDS-vCNC and hDS-vCNC samples. The peaks had similar appearances as reported for a homogeneously modified cellulose dissolved in D_2_O.^25^ However, the peaks of the ether linkers were slightly shifted in the electrolyte system used (Table S3). After modification, the signals for hemicellulose and the oxidized moieties were diminished, which suggests their preferential removal over solubilization during the reaction or workup. The presence of allyl glycidyl ether moieties in the diffusion-edited ^1^H NMR spectra, in which resonances of dissolved low molecular weight constituents are removed from the spectra,^46^ indicates that the functionalities are incorporated into a polymer. However, this can be attributed to both the anticipated covalent modification of CNCs and the formation of a physical mixture of CNCs and homopolymerized allyl glycidyl ether. In the ^1^H–^13^C HSQC spectra, no significant peaks for etherified glycopyranose units are apparent,^47^ which suggests only a minor direct modification of the cellulose backbone. To further exclude the possibility of the formation of a physical mixture, it was attempted to extract poly(allyl glycidyl ether) from the hDS-vCNC sample by stirring in DMSO-*d*_*6*_ for 16 h at 65 °C. The modified cellulose remained insoluble, and the obtained supernatant liquid only showed minor signals for low molecular weight allyl glycidyl derivatives (Figure S19). According to the diffusion-edited ^1^H NMR spectrum, no polymeric constituents were solubilized (Figure S20). In accordance with these observations, the NMR spectrum of the solid residue dissolved in the NMR electrolyte was identical with the nonextracted hDS-vCNC sample (Figure S24).

The results of the NMR characterization suggested that the isolated vCNC samples are primarily modified with covalently attached poly(allyl glycidyl ether) branches. The individual cellulose chains showed only minor signs of modification, which indicates that the attachment of further glycidyl moieties preferably occurred on already introduced side chains. Given the isolated yields, the observed removal of hemicelluloses and oxidized surface chains, and the known water solubility of cellulose homogeneously modified with allyl glycidyl ether, it seems plausible that CNCs with branched side chains can be isolated due to their insolubility in the chosen reaction medium. While the exact architecture of the grafted moieties on the cellulose surface chains is vital to understanding the impact on the cross-linking abilities of the modified fillers, further investigations will be conducted outside of this proof-of-concept study.

Additionally, quantitative ^1^H spectra were examined to estimate the amount of introduced vinyl moieties. Due to strong peak broadening in the viscous NMR samples and significant overlap of the relevant peaks with H_2_O, an aggressive baseline correction and external fitting of the peak areas had to be performed (see Supporting Information). Consequently, the obtained values must be treated as a rough estimate rather than a definite value. Integration and comparison of the C1–H region of the cellulose backbone and the CH_2_ peak adjacent to the vinyl groups suggested that the overall molar ratio of glycopyranose units to vinyl groups was approximately 10:1 in the lDS-vCNC sample and 10:5 in the hDS-vCNC sample.

**Figure 5:**
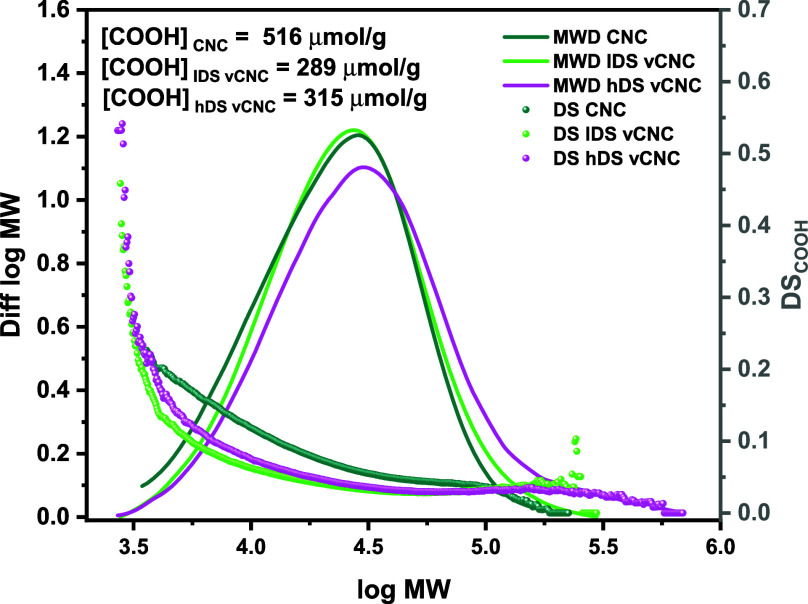
Normalized molar mass distribution plots obtained using the GPC-MALLS-RI setup (lines, diff log MW, left axis); DS_COOH_ determined by FDAM fluorescence labeling (scatter plot, right axis). Unmodified TO CNCs, lDS vCNCs (modified with low amounts of AGE), hDS vCNCs (modified with high amounts of AGE); estimated total concentration of COOH groups (top left, in μmol/g cellulose).

Further, the isolated CNC samples were analyzed using GPC-MALLS-RI to determine their molar mass distribution and their carboxyl content. The corresponding results are presented in Table 2 and Figure 5.

All samples exhibit unimodal distributions of the molecular weight in a range typical for HCl-hydrolyzed CNC samples.^48,49^ While the unmodified CNC and the lDS-vCNC samples are within a similar weight-average molecular weight (M_W_) range (29.5 and 32.7 kDa, respectively), the trace for the hDS-vCNC samples shows a shift to slightly higher molecular weight. Simultaneously, the recovery (calculated injected mass vs theoretically dissolved mass) for the unmodified CNCs and lDS-vCNCs is in the same range (0.175 mg and 0.159 mg), while the hDS-vCNC sample exhibits a 2-fold lower recovery (calculated mass: 0.086 mg). This indicates that the sample is partly lost during labeling and subsequent workup, presumably due to enhanced solubility of the highly modified moieties in the solvents used for activation and solvent exchange. Efforts to recover the dissolved chains by precipitation after labeling using ethanol or water were unsuccessful; instead, they remained in solution. This is in line with the reports from Qi et al.,^25^ who showed that ensuing full dissolution, the AGE-modified cellulose remained in solution even after washing and switching to pure water. This suggests that modified cellulose chains exhibiting higher solubility were leached out of the sample prior to analysis, leaving those chains that are less soluble due to a lower degree of modification, higher molecular weight, or lower charge. The molecular weight distribution of the remaining chains is accordingly slightly shifted to higher molecular weights. However, the desired modification of the cellulose chains with AGE oligomers would equally cause a shift in the molecular weight distribution. Based on the data available, no clear statement can be made about the origin of the MWD increase.

Determination of the COOH content and distribution was conducted using fluorescence labeling with FDAM. All determined values were far outside the calibration range (7.66–32.1 μmol g^–1^). Thus, applying the standard FDAM procedure^32^ can only give a rough quantitative estimation rather than absolute values. This becomes clear in comparison to the results of conductometric titrations of the individual samples. It was found that the unmodified CNCs exhibits the highest content of carboxyl groups for both FDAM labeling and conductometric titration. After modification, the carboxyl content is reduced for both lDS-vCNCs and hDS-vCNCs. This is in line with the previously discussed trends of the dissolution of highly modified chains as well as a mass dilution effect caused by grafted moieties. Furthermore, the results obtained from FDAM labeling and conductometric titrations (see Figure S4) show the same trends of decreasing specific charge with an increasing amount of modifying agent.

In all samples, the COOH group content was highest in the low molecular weight range. The COOH distribution changes upon modification with a decrease in the COOH concentration, especially in the low molecular weight range. This observation is in line with the hypothesis that the charged cellulose chains, which are situated on the nanocrystal surface and therefore exposed the most to modification, become more hydrophilic and desorb from the crystal. Further, chain scission due to β-elimination degradation reactions lowers the molecular weight of the charged chains.^43^ As a consequence, the COOH concentration for the modified samples is significantly increased in the low molecular weight range.

**Figure 6:**
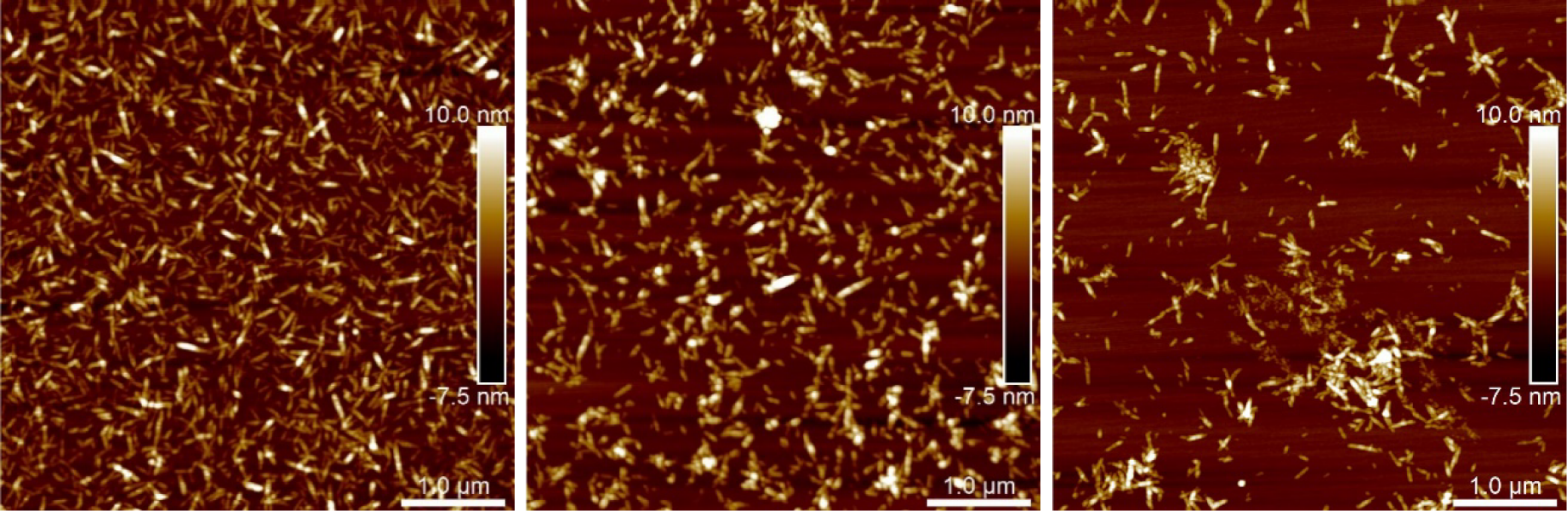
AFM images of the nanocellulose particles before (left) and after (top) modification with low (middle) and high (right) amounts of allyl glycidyl ether.

The AFM images (Figure 6) show that the cellulose particles retain their general morphology, while differences in their aspect ratio are within the range of the standard deviation (see Figure S5 and Table S1). This can be attributed to the degradation of the cellulose in the reaction medium as well as the surface modification, which makes the particles softer and thereby changes the interactions with the probe. It is also noticeable that the tendency to aggregate increases with the increasing amount of AGE in the modification reaction.

**Figure 7:**
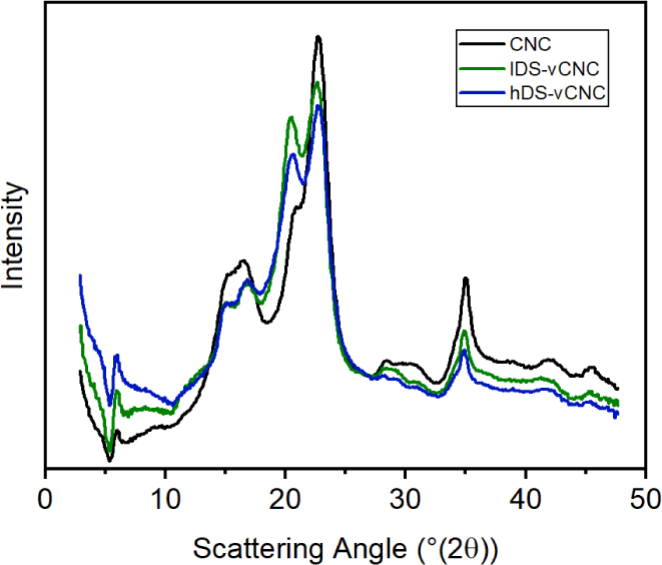
WAXS diffractograms of the unmodified and modified CNCs.

The WAXS diffractograms (Figure 7) show that the modified particles are still crystalline, even though the signal-to-noise ratio appears to decrease. This is to be expected due to mass dilution effects of the amorphous grafted side chains as well as the swelling of the cellulose in the basic reaction medium, which reduces the domain size of the crystallites, but it is as likely that the swelling of the cellulose in the reaction medium as well as the increased solubility results in increased desorption of the surface chains from the particles and therefore permanently reduced crystallinity. Crucially, the intensity ratios of the observed reflexes are changing in line with an ongoing transition from cellulose I toward cellulose II.^50^ With 6 wt % of sodium hydroxide, the reaction medium is shy of the conditions required for mercerization, but both surface charges and grafted epoxy moieties increase the solubility of the chains and are thus expected to ease the swelling of the crystallites.

### Mechanical Testing

**Figure 8:**
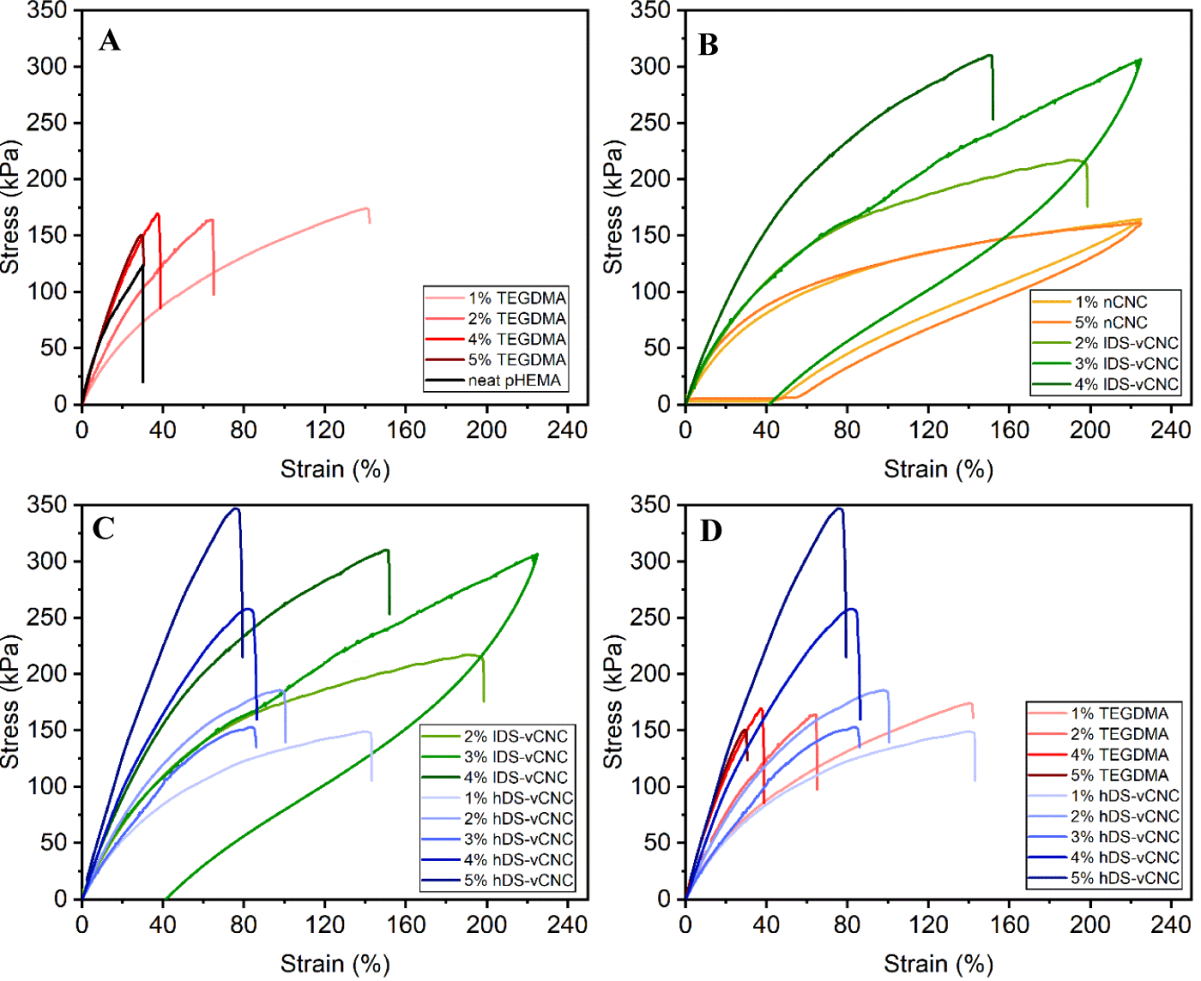
Stress–strain curves of polymer specimen of neat and TEGDMA-cross-linked pHEMA (A), polymer composites containing unmodified and modified CNCs (B), vCNC-cross-linked polymer composites containing CNCs with low and high degrees of vinylation (C), and TEGDMA-cross-linked and hDS-vCNC cross-linked polymer composites (D).

To assess the feasibility of using the vinylated nanocellulose as a comonomer in a radical polymerization reaction, we synthesized pHEMA-*co*-vinyl-CNC polymer hydrogels *via* free radical polymerization of the aqueous CNC dispersions in 50 wt % HEMA. While the vinyl groups on the CNCs were acting as cross-linking agents for copolymerization, the remaining carboxylic groups were deemed to be inert in the polymerization reaction. Furthermore, we compared these CNC-containing samples to the ones containing a typical molecular cross-linker TEGDMA. Stress–strain curves of the polymer hydrogels obtained from these polymerization reactions are shown in Figure 8.

As expected, the integration of up to 5 wt % of cross-linking monomers into the polymer hydrogel increases the yield strength and tends to increase the stiffness, as shown in Figure 8A. Numerical values for ultimate strength and strain, stiffness, and toughness obtained from the curves shown in Figure 8 are listed in Table S2.

Counterintuitively, in the case of the samples containing 1 and 2 wt % TEGDMA, the stress at failure is increased, but the stiffness of the compounds is reduced. This suggests an increased flow of the polymer chains, which dissipates the energy, inducing stress on the material. A possible cause for this behavior might be the gel effect, which in turn influences the polymerization kinetics and, therefore, the glass transition temperatures, which will be discussed in more detail below. The introduction of sufficient amounts of cross-linker negates this reduction in stiffness and leads to the expected outcome of increased yield strengths.

These tendencies are mirrored for the compounds containing pHEMA with unmodified and vinylated CNCs as fillers. Here, the strain at failure is increased from 30% for the neat pHEMA compound to 220% for the composite containing unmodified CNCs. This is in line with the well-established concept that the matrix–filler interactions in nanocomposites enable a more efficient distribution of the load applied to the material, which reduces the chance of crack formation and thereby strengthens the material.^12,51^ Meanwhile, the measured stresses are reduced as well, indicating an effect of the fillers on the polymerization kinetics. Again, this might be due to the increased viscosity of the reaction mass in the presence of the fillers, but CNCs have been reported to scavenge radicals in polymerization reactions, thus directly interfering with the polymerization process. Beyond considering the polymer itself, in the presence of one-dimensional fillers, energy can be dissipated by aligning the fillers with the direction of applied stress. This process is hindered, however, in the case of the vinylated CNC fillers. With the reactive surface groups binding polymer chains to the particle surface and increasing the degree of cross-linking, far stronger interactions between filler and matrix are achieved. Consequently, the alignment of the filler particles requires far more energy. Therefore, as shown in Figure 8B, the cross-linking of the polymer by reactive surface groups on the filler particles significantly increases the stiffness and toughness of the composites. As seen before in the case of monomeric cross-linkers, however, the strains at failure are lower for higher degrees of cross-linking.

This trend continues for CNC fillers with a higher degree of surface vinylation. With the introduction of more cross-linking points of the filler surfaces, the materials become stiffer still, but at the expense of reduced strains at failure. As such, incorporating 5 wt % of highly vinylated CNC filler particles reduces the strain at failure to 80%. At this point, it can be assumed that the cross-linking effect dominates the final properties, not the impact of the fillers on viscosity and thus the polymerization kinetics.

Comparing the highly vinylated CNCs with the monomeric cross-linker (see Figure 8D), it is obvious that both strategies achieve similar results and tendencies with regard to the strain at failure. This similar performance of both cross-linking agents is especially obvious in the results obtained for 1 wt % monomeric cross-linker and 1 wt % hDS-vCNCs. Increasing the concentration of either cross-linking agent is reflected in increased toughness, while for the vinyl-CNCs, the nanofiller effect of preventing crack formation yields additional performance with regard to the strains at failure.

**Figure 9:**
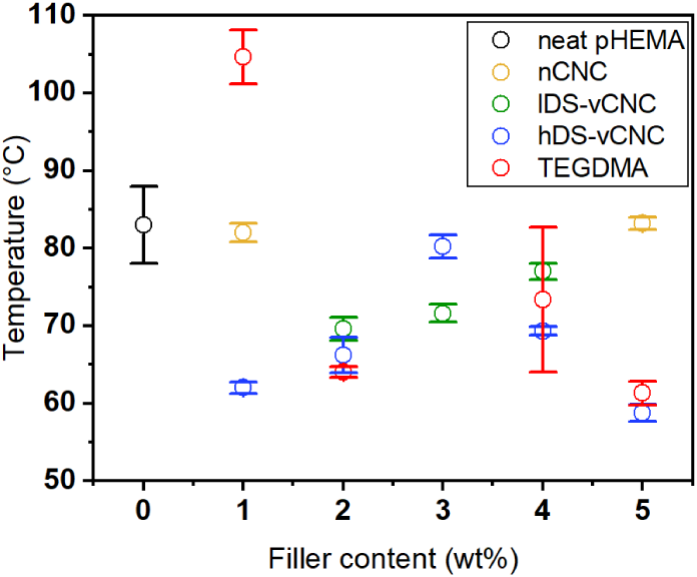
Glass transition temperatures determined by DSC for the composite materials.

For the vinylated CNCs, these trends translate to the ultimate stresses as well, although global trends remain absent. This could be due to differences in polymer architecture, as illustrated by the glass transition temperatures in Figure 9.

The glass transition temperatures we observed for our materials range from 60 to 110 °C. A wide range of glass transition temperatures for pHEMA has been reported in the literature, with reported temperatures between 80 and 120 °C.^21,52,53^ The discrepancies in the glass transition temperatures have been attributed to the polymer tacticity and the polymerization medium. As such, syndiotactic pHEMA has been reported to have a glass transition temperature of 120 °C, while atactic pHEMA already goes through glass transition at 80 °C.^52^ Additionally, it has been observed that free radical polymerization of HEMA in the presence of increasing amounts of water leads to a decrease in the glass transition temperature as well.^53^ This has been attributed to a disruption in the network of hydrogen bonds between the 2-ethyl-side chains by water, which acts as a plasticizer.^54^ Both factors are reflected in our experiments. The glass transition points of most of our polymers are slightly below 80 °C, which is to be expected for atactic pHEMA polymerized in the presence of significant amounts of water. The highest glass transition temperature was found for small amounts of monomeric cross-linker, which is in line with the formation of a loosely cross-linked network, which has minimal impact on viscosity changes compared to the polymerization of neat pHEMA. Adding fillers to the reaction medium or increasing the cross-linker concentration, however, resulted in a reduction of the glass transition temperature. Swelling the polymers in DMSO followed by extraction with ethanol and subsequent drying in vacuo did not have an impact on the glass transition, indicating that plasticization by water or remaining monomers or oligomers did not have a significant impact in this context (see Figure S7). Rather, it must be concluded that it is the polymer architecture that gives rise to these properties. Unfortunately, the far-from-self-evident behavior of the glass transition temperatures also illustrates that the mechanical and rheological behavior is impacted not only by the composition of the specimens but also by the polymerization process itself.

## Conclusions

In this paper, we have demonstrated a successful route toward surface vinylation of cellulose nanocrystals. The presence of vinyl bonds has been verified by FTIR, Raman, and solution-state NMR spectroscopy, while we could show the retention of the particles’ crystallinity by means of WAXS experiments. Given the choice of an aqueous modification route and long exposure to high pH during the modification process, side reactions and degradation processes exerted a toll on our yields. Thorough characterization of the modified celluloses showed the presence of polymeric vinyl moieties. However, in comparison to the homogeneously modified congeners, no direct spectroscopic evidence of strong covalent modification of the cellulose backbone could be obtained. Nonetheless, the unimodal molecular weight distributions and the inability to extract the vinyl-containing polymers with H_2_O or DMSO do not support an only physical mixture of CNCs with poly(AGE). Overall, we suggest that the modification is primarily present in longer side chains, covalently attached to the cellulose surface. With the prepared materials, we successfully proved the concept of using vinylated cellulose nanocrystals as cross-linking filler particles in free radical polymerizations of HEMA, obtaining polymer composites with superior performance to the homopolymer and HEMA/TEGDMA elastomers. Given that the polymerization kinetics seemed to be affected by the modified nanofillers, subsequent studies will need to focus on fine-tuning the reaction parameters.

## Supporting Information Available

Schematic drawings of the used Teflon polymerization molds, picture of the molds in use after finished polymerization, calculations of cross-sectional areas of polymer specimens, conductometric titration curves of neat and modified CNCs, dimensions of the neat and modified CNCs, summary of the mechanical properties of the produced polymer composites, DSC traces after swelling and extraction, NMR spectroscopy data including peak assignments, results from prescreening with bacterial cellulose, spectral catalog for modified wood CNCs, and peak fitting for vinyl content assessment (PDF)
