# Supplementary material for: Surface-Vinylated Cellulose Nanocrystals as Cross-Linkers for Hydrogel Composites
Source: Biomacromolecules. 2025 Mar 11;26(4):2282–92. doi: 10.1021/acs.biomac.4c01619 (PMC12004536; doi:10.1021/acs.biomac.4c01619)
Supplement: Supplementary file 1 — bm4c01619_si_001.pdf [file bm4c01619_si_001.pdf]

Supporting Information

Surface-vinylated cellulose nanocrystals as cross-linkers for hydrogel composites

Marcel Kröger[1], Timo Pääkkönen[1,2], Lukas Fliri[1], Anna F. Lehrhofer[3], Irina Sulaeva[3,4], Antje Potthast[2], Eero Kontturi[1]*

1) Department of Bioproducts and Biosystems, Aalto University, FI-00076 Aalto, Finland

2) Nordic Bioproducts Group Oy, Tietotie 1, 02150 Espoo, Finland

3) Institute of Chemistry of Renewable Resources, Department of Chemistry, University of Natural Resources and Life Sciences, Vienna (BOKU), Muthgasse 18, A-1190 Vienna, Austria

4) Core Facility "Analysis of Lignocellulosics" (Alice), University of Natural Resources and Life Sciences, Vienna (BOKU), Konrad-Lorenz Strasse 24, A-3430 Tulln, Austria

Contents

Polymerization mold S3

Calculation of cross-sectional areas for slightly concave particles S4

Conductometric titrations of neat and modified CNC S6

Particle dimensions of the neat and modified CNC S7

Mechanical properties of the polymer samples S8

DSC traces after swelling and extraction S10

NMR spectroscopy S11

Prescreening with bacterial cellulose S12

Spectral catalogue S16

Peak fitting and calculation of vinyl content S28

References S32

Polymerization mold

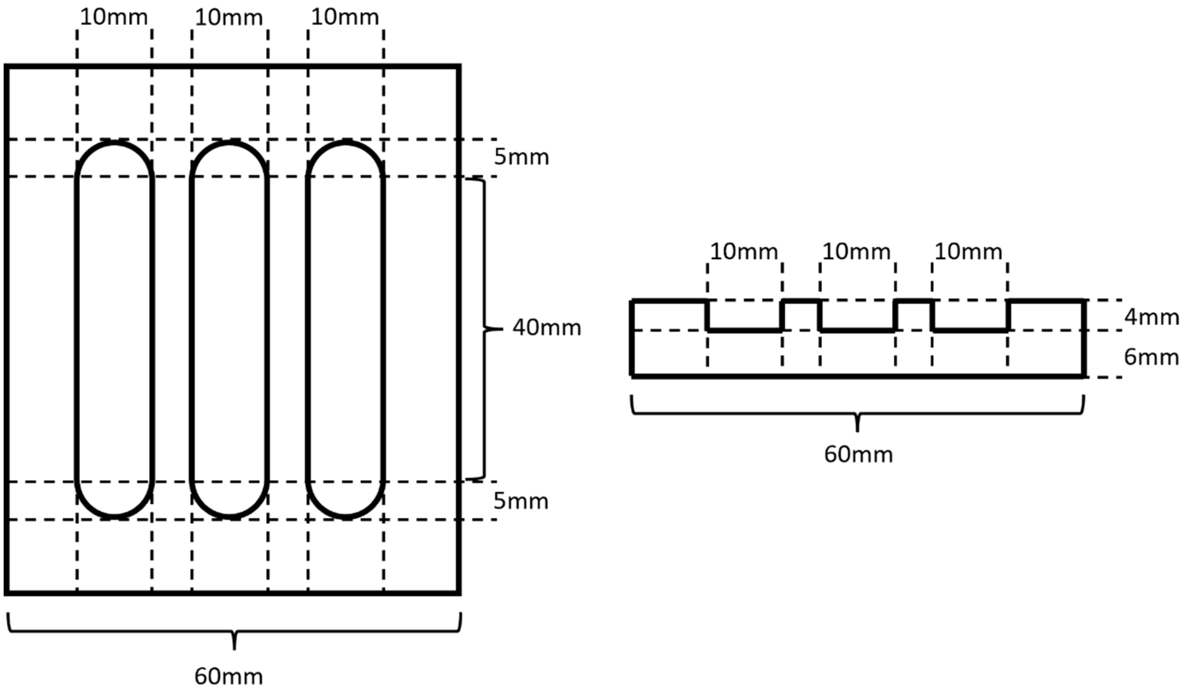

Figure S1. 1:1 drawing of the molds used for the polymerization reactions.

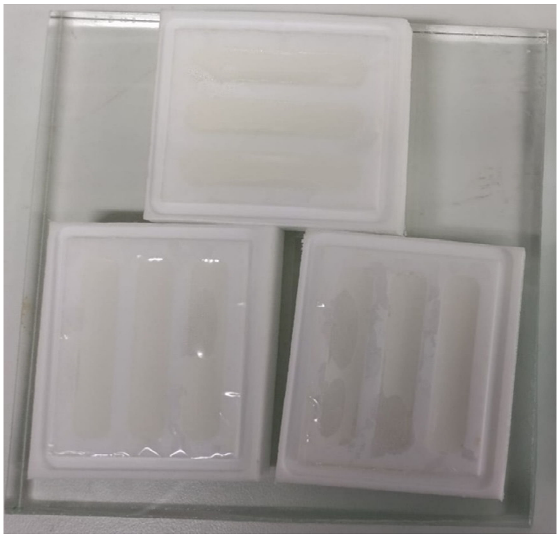

Figure S2. Composite samples after the polymerization reaction in teflon molds covered with PE-film.

Calculation of cross-sectional areas for slightly concave particles

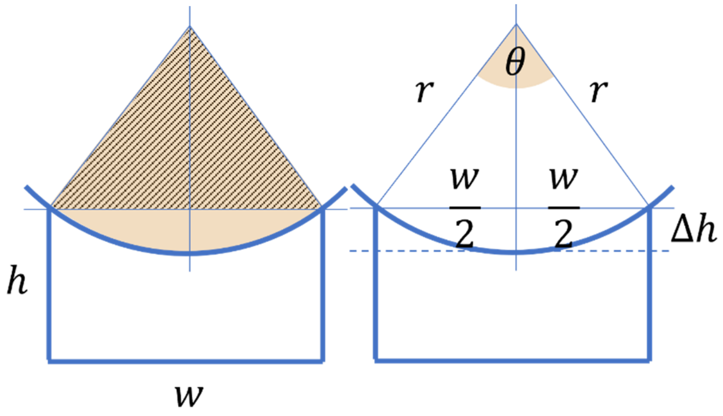

Figure S3. Exaggerated profile of the produced specimens to illustrate the slightly concave surface. By assuming that the shape is circular rather than ellipsoid, the radius of curvature can be calculated from the height difference between the center of the cross section and its edges, allowing to calculate the area of the void in the cross section compared to a rectangle.

The cross-sectional area can be calculated according to equations (S 1) and (S 2):

$$A = wh - A_{gap} \tag{S 1}$$

$$A_{gap} = \pi r^2 \frac{\theta}{2\pi} - \frac{w}{2}(r - \Delta h) \tag{S 2}$$

As such, the radius of curvature r and the angle Θ are needed to proceed. Both can be found as follows:

Expressing the surface curvature as a function of the radius yields equations (S 3), (S 4) and (S 5):

$$r^2 = x^2 + (y - r)^2 \tag{S 3}$$

$$r^2 = \left(\frac{w}{2}\right)^2 + (\Delta h\ - r)^2 \tag{S 4}$$

$$(r - \Delta h) = \frac{w^2 - 4\Delta h^2}{8\Delta h} \tag{S 5}$$

Based on these expressions, the angle Θ can be calculated according to equation (S 6):

$$\theta = 2arctan \frac{\frac{w}{2}}{(r - \Delta h)} \tag{S 6}$$

$$= 2arctan \frac{4w\Delta h}{(w^2 - 4\Delta h^2)}$$

Combining equations (S 2) through (S 6) yields the expression for the area of the cross-sectional area (equation (S 7)):

$$A = wh - r^2 arctan \left(\frac{4w\Delta h}{(w^2 - 4\Delta h^2)}\right) - \left(\frac{w^3 - 4w\Delta h^2}{8\Delta h}\right) \tag{S 7}$$

Conductometric titrations of neat and modified CNC

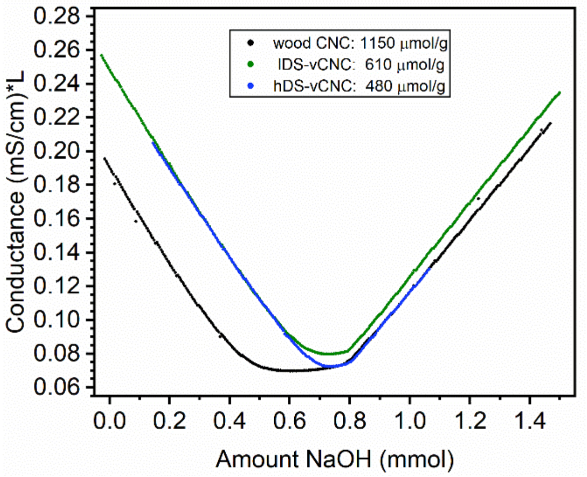

Figure S4. Titration curves of the used CNC.

Particle dimensions of the neat and modified CNC

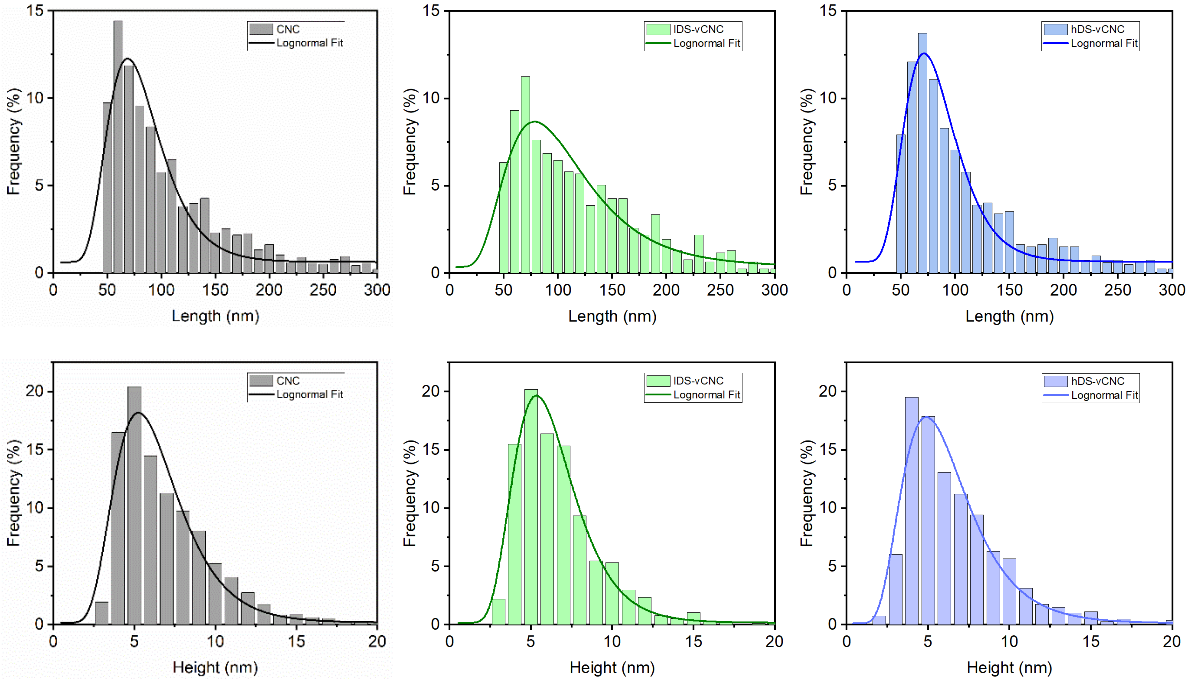

Figure S5. Length and height distribution of the original and the surface-modified CNC based on AFM analysis.

Table S1. Mean particle lengths and heights based on Lognormal fitting of the distributions in Figure S5.

	Height (nm)	Length (nm)	Aspect ratio (nm)
CNC	6.5 ± 2.5	83 ± 30	13 ± 10
lDS-vCNC	6.4 ± 2.2	109 ± 54	17 ± 15
hDS-vCNC	6.3 ± 2.7	84 ± 28	13 ± 10

The analyzed particles mostly retained their size throughout the modification routine despite the images getting progressively blurrier. This may suggest that the particles retain their size or that the added surface groups are too soft to be resolved here. The only major change is seen in the length of the particles with a lower degree of surface vinylation. This may be indicative of a transition from charge stabilization to steric stabilization.

Mechanical properties of the polymer samples

Table S2. Values for glass transition temperatures, Strains and stresses at failure, as well as stiffness and toughness of the considered polymer samples.

	Glass transition	Strain at Failure	Stress at Failure	Initial Stiffness*	Toughness
	°C	%	kPa	Mpa	kJ/m^3
neat pHEMA	83	30	121	46	22
1% TEGDMA	104	141	174	48.1	161
2% TEGDMA	64	64	163	62.1	65
4% TEGDMA	73	37	170	89.8	39
5% TEGDMA	61	29	151	75.8	27
1% nCNC	82	>225	>164	46	268
5% nCNC	83	>225	>161	65.7	272
2% lDS-vCNC	70	192	217	44.8	311
3% lDS-vCNC	72	>225	>307	63	428
4% lDS-vCNC	77	150	310	65.3	313
1% hDS-vCNC	62	141	149	38.3	149
2% hDS-vCNC	66	98	186	51.8	125
3% hDS-vCNC	80	84	153	47.8	84
4% hDS-vCNC	69	82	258	59.4	139
5% hDS-vCNC	59	74	347	69.7	165

The measured stress-strain curves had no linear regime to draw elastic moduli from. Therefore, we evaluated the initial stiffness of the samples.

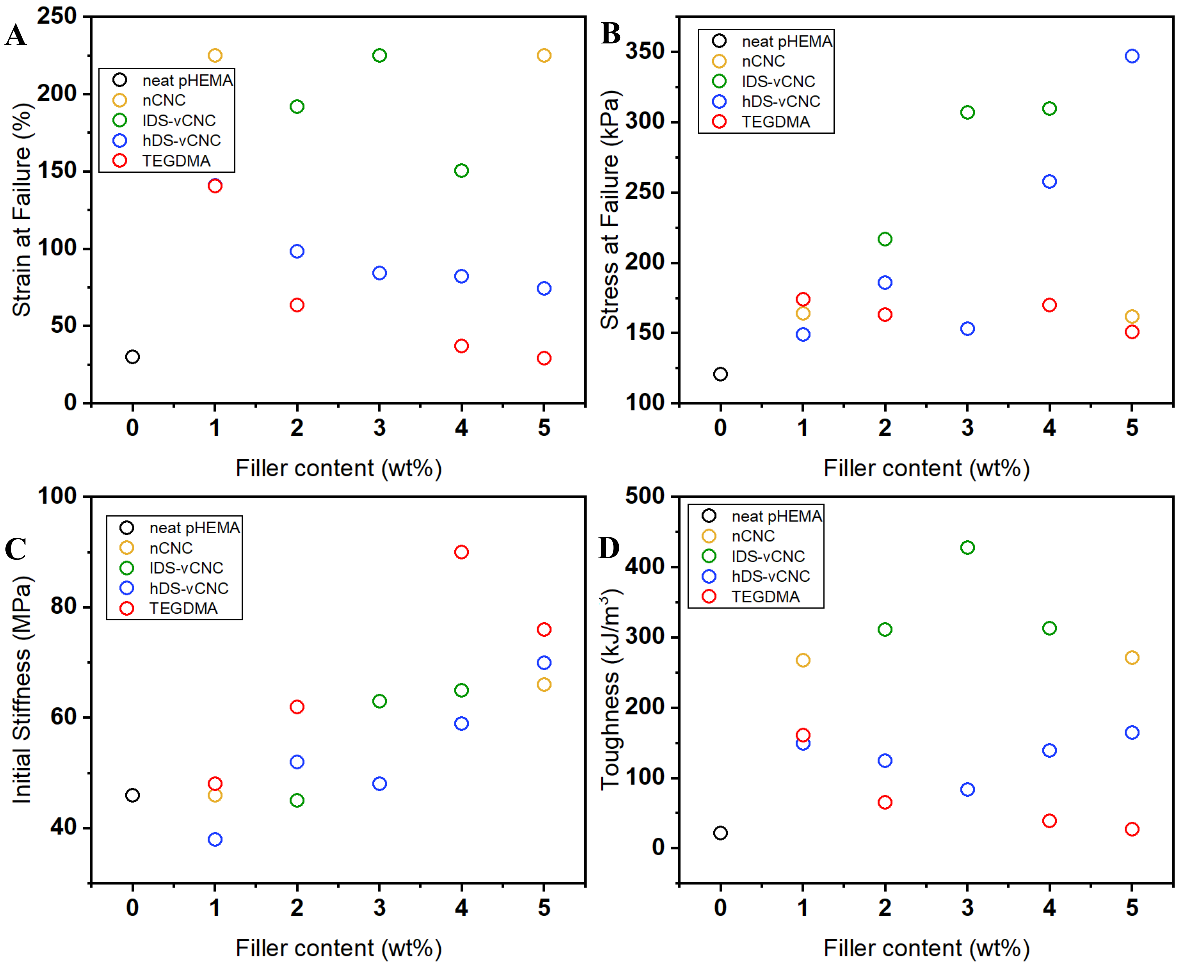

Figure S6. Graphic representation of the impact of filler content on the values for Strain at Failure (A), Stress at Failure (B), initial stiffness (C) and toughness (D) displayed in **Table S2**.

DSC traces after swelling and extraction

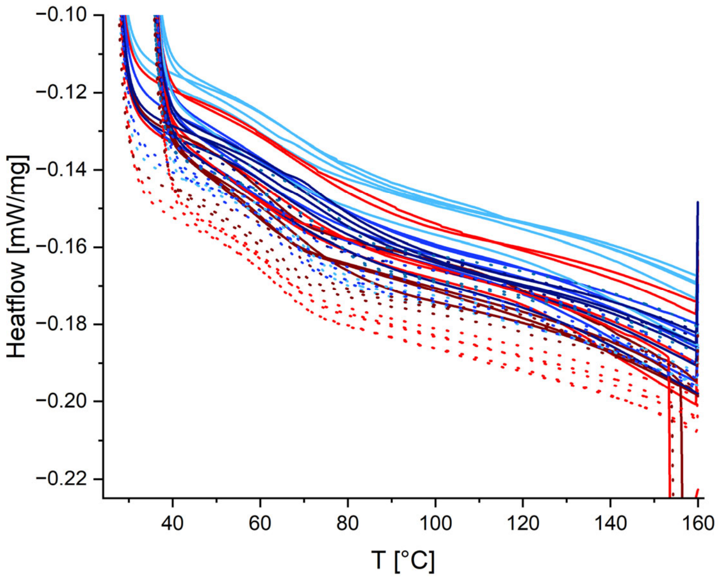

Figure S7. DSC traces of washed polymer samples (solid lines) compared to the previous results (dotted).

All curves are shifted towards lower heat flow, *i.e.*, lower thermal capacity. Nevertheless, across the board no significant changes in the glass transition are observed.

NMR spectroscopy

Peak assignments:

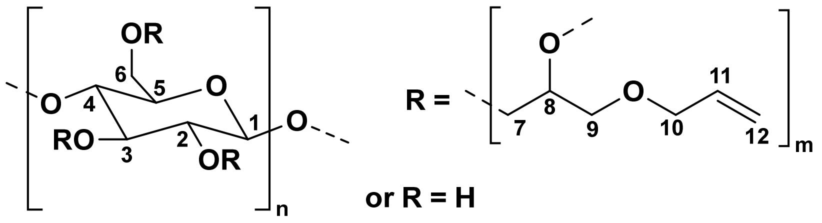

Scheme S1. Chemical structure of the prepared vCNCs and numbering of the different moieties for NMR assignment (see Table S3). Note: Based on the NMR characterization it is assumed that the derivatization is mainly present in longer allyl glyceryl ether residues and that the modification of individual glucopyranose units is rather small overall. However, it was not possible to determine the degree of substitution. Furthermore, no full spin systems of anhydroglucuronic acid groups could be distinguished, which is why we neglect those moieties in the assignments.

Table S3. Peak assignments for the vCNC samples prepared in this study, compared to the homogenously modified celluloses reported by Qi et al. [1] Differences in the peak shifts can be attributed to differences in the NMR solvents and measuring temperatures.

Nr.	Moiety	P_{4444}[OAc]:DMSO-d_6 (1:4 wt%); 65 °C[a]		D_2O; temperature not stated	
		^{1}H	^{13}C	^{1}H	^{13}C
1	CH	4.40	102.3	4.41	102.1
2	CH	3.05	72.9	3.24	73.1
3	CH	3.42	72.4	3.54	74.8
4	CH	3.35	78.3	3.61	78.4
5	CH	3.24	75.1	3.44	74.8

6	CH_2	3.68	59.9	3.68	60.1
7[b]	CH_2	3.36	71.5	3.49	70.7
8	CH	3.75	68.0	3.90	69.0
9[b]	CH_2	3.42	72.4	3.54	74.0
10	CH_2	3.95	70.7	3.97	71.9
11	CH	5.87	134.9	5.85	134.3
12	CH_2	5.09 / 5.20	115.0	5.20	118.1

[a] assignments based on the multiplicity edited ^{1}H-^{13}C HSQC spectrum of hDS-vCNC.

[b] due to peak superposition and splitting the CH_2 groups could not be properly differentiated. Most intensive peak in the respective peak area was given.

Prescreening with bacterial cellulose

The used reaction conditions were initially optimized for CNCs prepared by TEMPO oxidation of bacterial cellulose hydrolyzed with HCl gas [2]. Different BC-vCNCs were prepared and purified following the protocol described in the main manuscript. The scale of the reaction and proportion of used starting materials was varied (**Table S4**) while the reaction time was kept constant at 72h. Influences of the reaction conditions on the introduction of vinyl moieties and their reproducibility were tentatively screened using diffusion edited ^{1}H NMR spectroscopy.

Table S4. Reactant amounts used in the prescreening experiments on bacterial cellulose. Equivalents (eq) refer to the amount of glucopyranose units (AGU) in the cellulose.

**Entry**	**Bacterial cellulose**				**NaOH**			**AGE**		
	**Suspension**	**wt%**	**Dry**	**n(AGU)**	**Mass /**	**n(NaOH)**	**eq.**	**Mass /**	**n(AGE)**	eq.

	/ g		mass/ g	/ mmol	g	/ mmol		g	/ mmol	
**vBC-1**	14	7.5	1.1	6.5	0.7	16.4	2.5	4.1	35.6	5.5
**vBC-2**	14	7.5	1.1	6.6	0.7	16.6	2.5	4.0	35.4	5.3
**vBC-3**	100	7.5	7.5	46.3	5.1	126.9	2.7	4.0	35.4	0.8
**vBC-4**	100	7.5	7.5	46.3	5.1	126.5	2.7	15.5	136.0	2.9
**vBC-5**	94	10.0	9.4	58.2	6.8	169.5	2.9	37.7	330.3	5.7

The comparison of the diffusion edited ^{1}H spectra of **vBC-1** with **vBC-2** showed that conducting the reaction under identical reaction conditions resulted in the introduction of a similar amount of vinyl moieties (**Figure S8**).

In the trials at higher concentrations the equivalents of ally glycidyl ether (AGE) added to the reaction mixture were varied. The incorporation of vinyl moieties could be slightly increased (**Figure S9**). However, there was no direct relationship between the amount of AGE and covalently attached side chains. *I.e.*, using six times the amount of AGE does not increase the vinyl peak areas by a factor of six. For **v-BC-5**, the material prepared under the harshest conditions, solubility issues and significant peak broadening were encountered during the NMR measurements. This is likewise ascribable to more severe crosslinking, serving as a potential unwanted side reaction under harsher conditions.

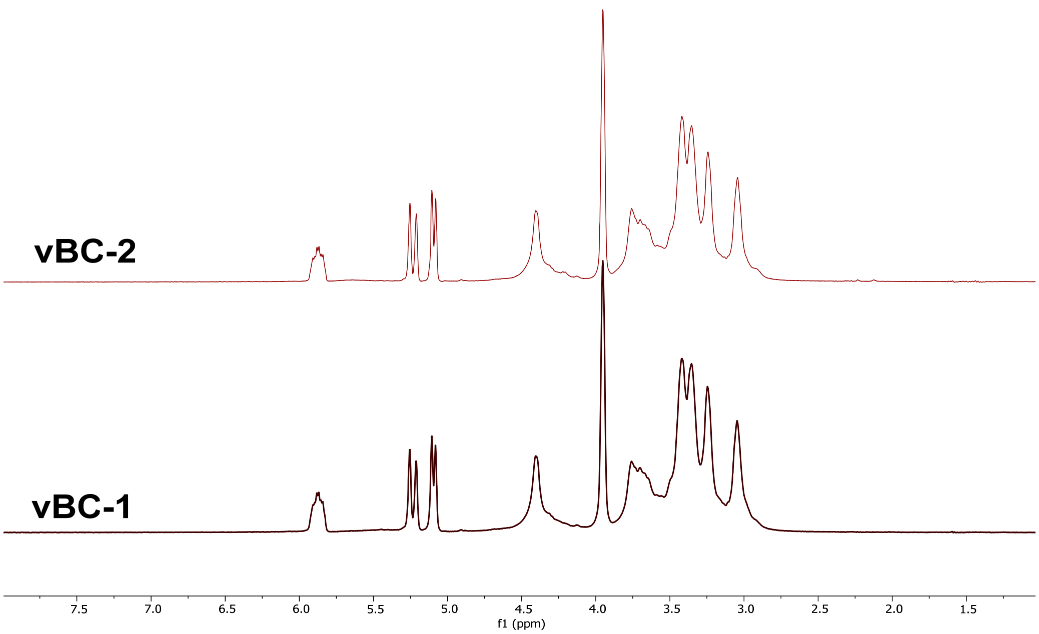

Figure S8. Comparison of the diffusion edited ^{1}H NMR spectra ([P_{4444}][OAc] : DMSO-d_6 (1:4); 400 MHz; 65°C) of **vBC-1** (5 wt%. **bottom**) and **vBC-2** (5 wt%. **top**). Both samples were prepared using the same reaction conditions and resulted in an almost identical introduction of vinyl moieties.

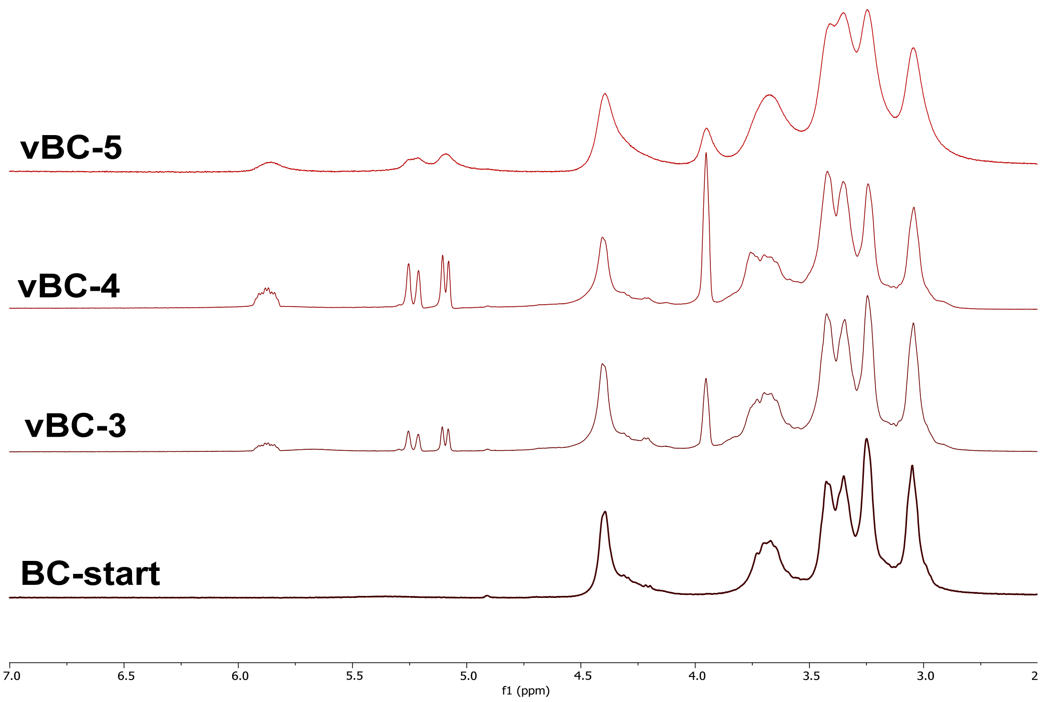

Figure S9. Comparison of the diffusion edited ^{1}H NMR spectra ([P_{4444}][OAc] : DMSO-d_6 (1:4); 400 MHz; 65°C) of the bacterial cellulose starting material (**BC start**) with vBCs prepared with increasing amounts of AGE (**Table S4**). All samples were measured at a concentration of 5 wt%. **vBC-5** was partially insoluble in the electrolyte system.

Spectral catalogue

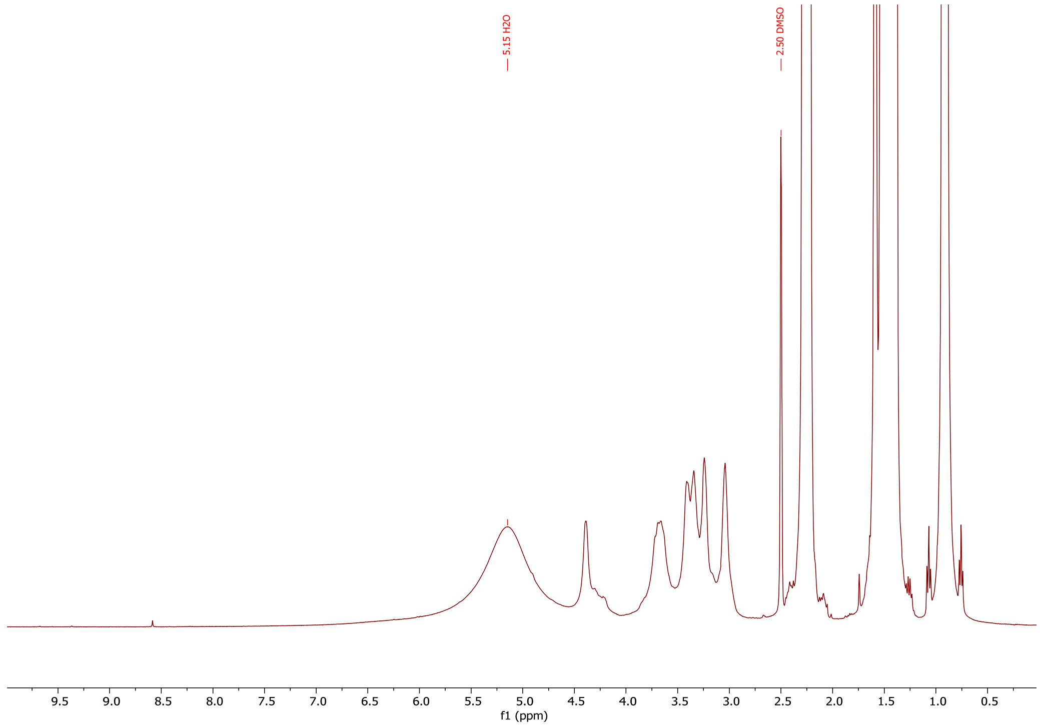

Figure S10. Quantitative ^{1}H NMR spectrum ([P_{4444}][OAc] : DMSO-d_6 (1:4); 400 MHz; 65°C) of the TEMPO oxidized wood CNC starting material (5 wt%). Full spectral area with electrolyte and water resonances is shown.

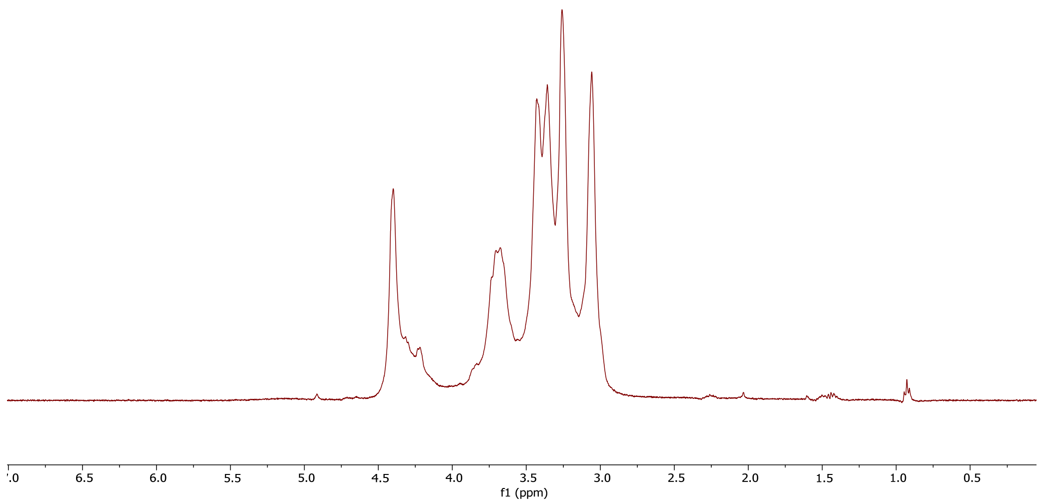

Figure S11. Diffusion edited ^{1}H NMR spectrum ([P_{4444}][OAc] : DMSO-d_6 (1:4); 400 MHz; 65°C) of TEMPO oxidized wood CNC starting material (5 wt%).

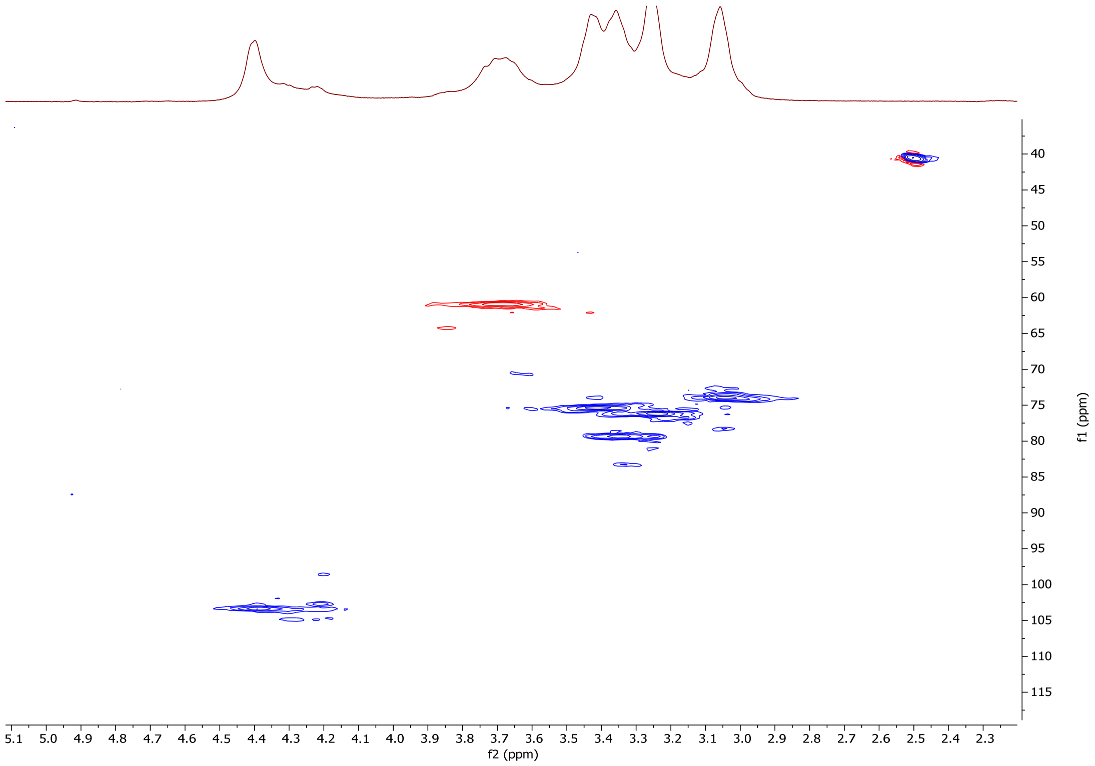

Figure S12. Multiplicity-edited HSQC spectrum ($[P_{4444}][OAc]$:DMSO-d_6 (1:4); 400 MHz ^1H frequency; 65°C) TEMPO oxidized wood CNC starting material (5 wt%). CH_2 resonances are shown in red. CH / CH_3 signals are shown in blue. On top the diffusion edited ^1H spectrum is inserted. Only the zoom into the polysaccharide region is shown.

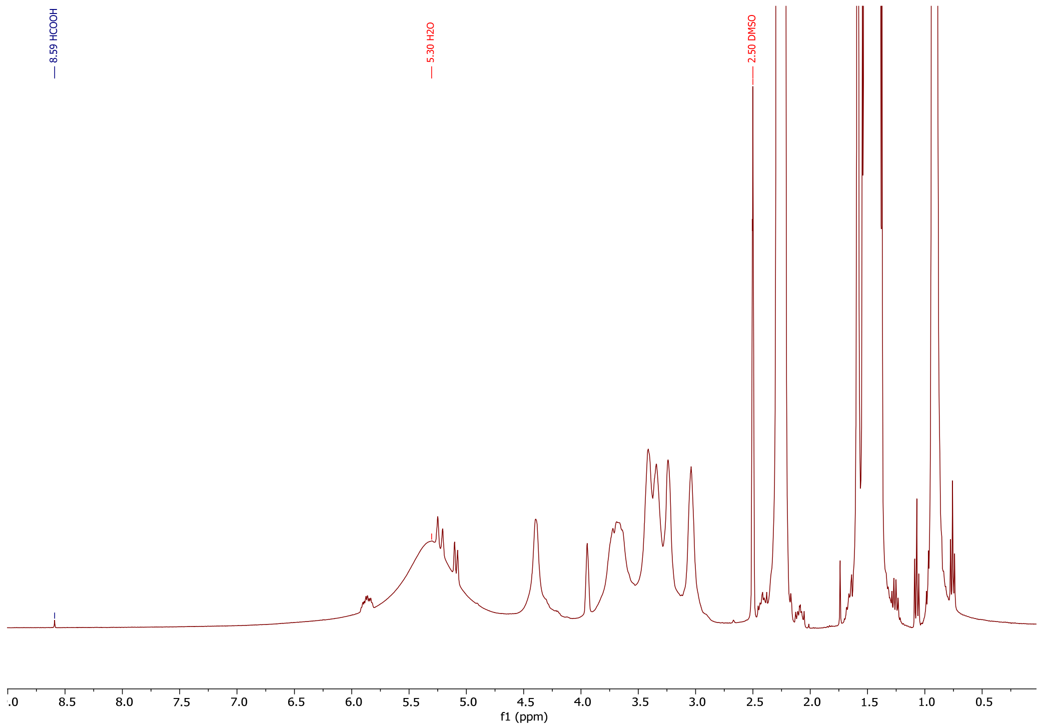

Figure S13. Quantitative ^1H NMR spectrum ($[P_{4444}][OAc]$: DMSO-d_6 (1:4); 400 MHz; 65°C) of the lDS-vCNC sample (5 wt%). Full spectral area with electrolyte and water resonances is shown.

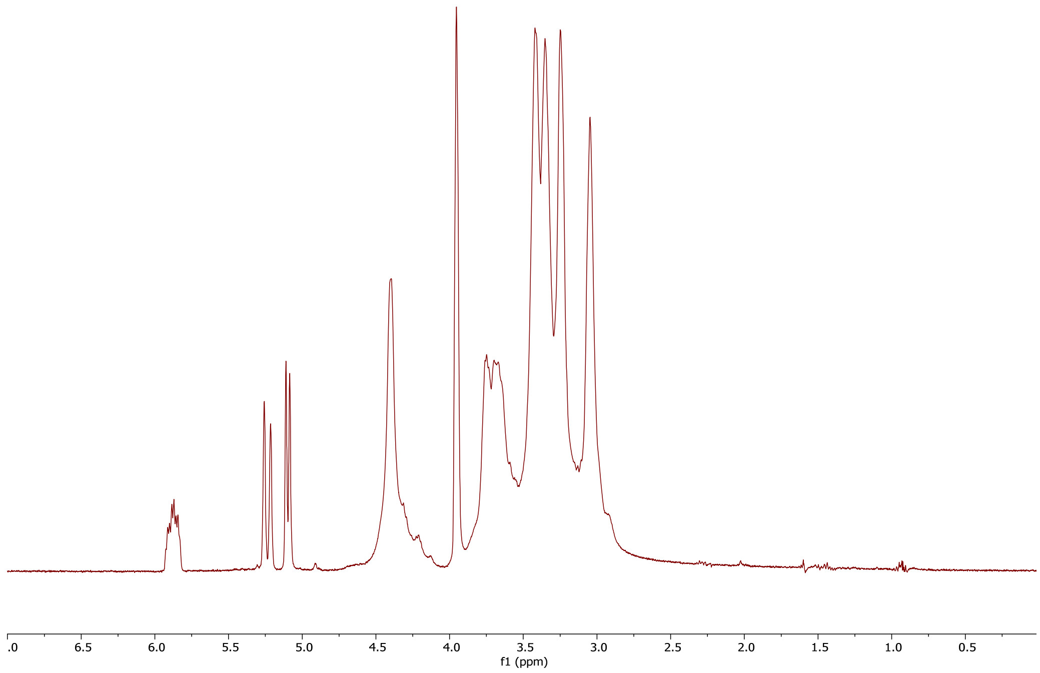

Figure S14. Diffusion edited ¹H NMR spectrum ([P_{4444}][OAc] : DMSO-d_6 (1:4); 400 MHz; 65°C) of the lDS-vCNC sample (5 wt%). Full spectral area is shown.

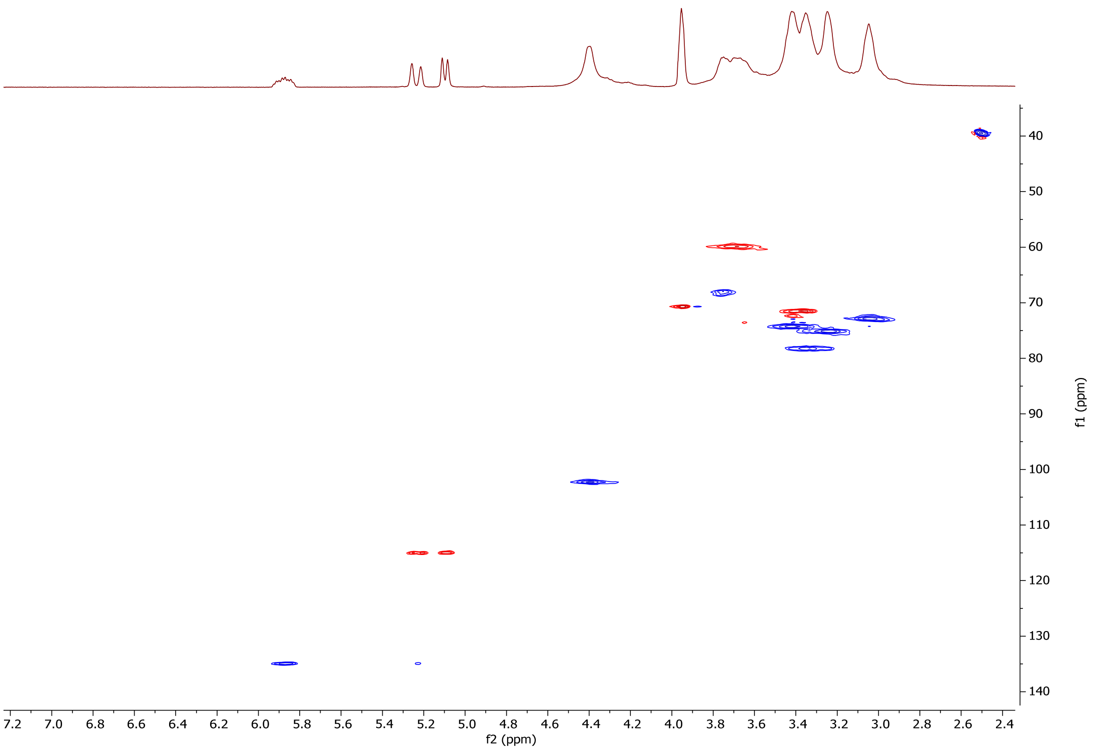

Figure S15. Multiplicity-edited HSQC spectrum ([P_{4444}][OAc] : DMSO-d_6 (1:4); 400 MHz ^1H frequency; 65°C) of the lDS-vCNC sample (5 wt%). CH_2 resonances are shown in red. CH / CH_3 signals are shown in blue. On top the diffusion edited ^1H spectrum is inserted. Only the zoom into the polysaccharide region is shown.

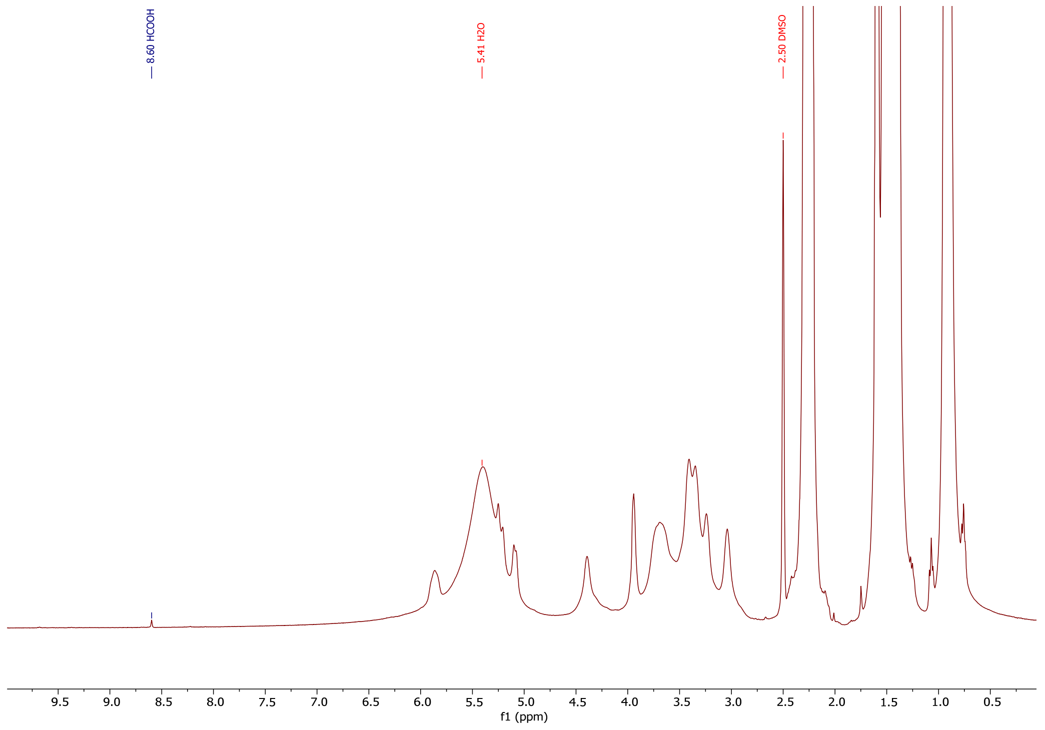

Figure S16. Quantitative ^1H NMR spectrum ([P_{4444}][OAc] : DMSO-d_6 (1:4); 400 MHz; 65°C) of the hDS-vCNC sample (5 wt%). Full spectral area with electrolyte and water resonances is shown.

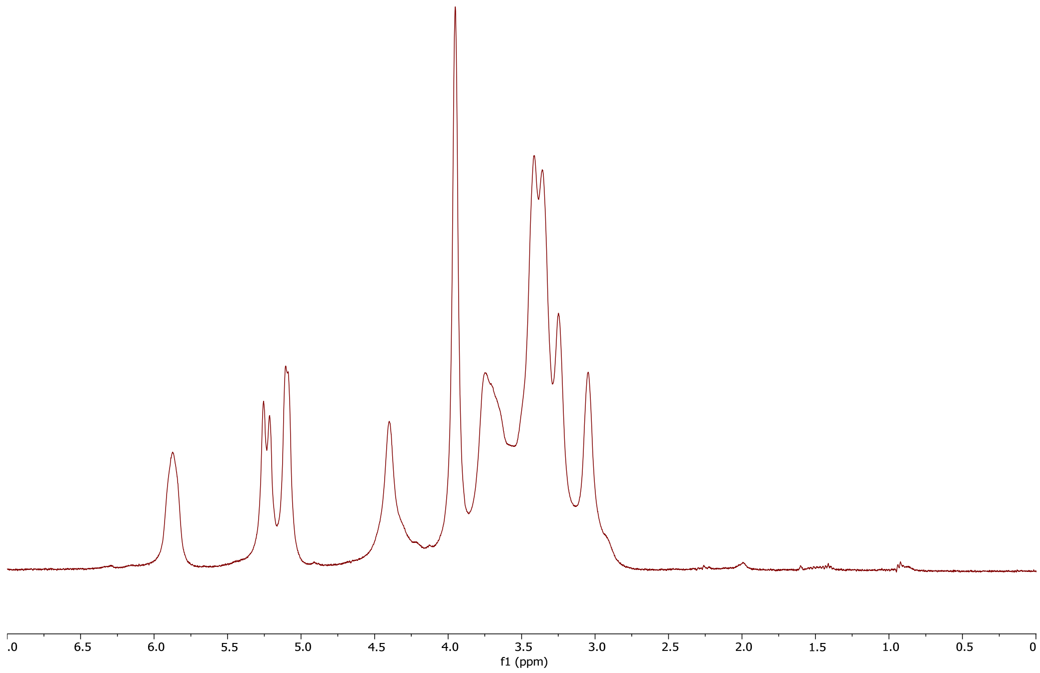

Figure S17. Diffusion edited ^{1}H NMR spectrum ([P_{4444}][OAc] : DMSO-d_6 (1:4); 400 MHz; 65°C) of the hDS-vCNC sample (5 wt%)

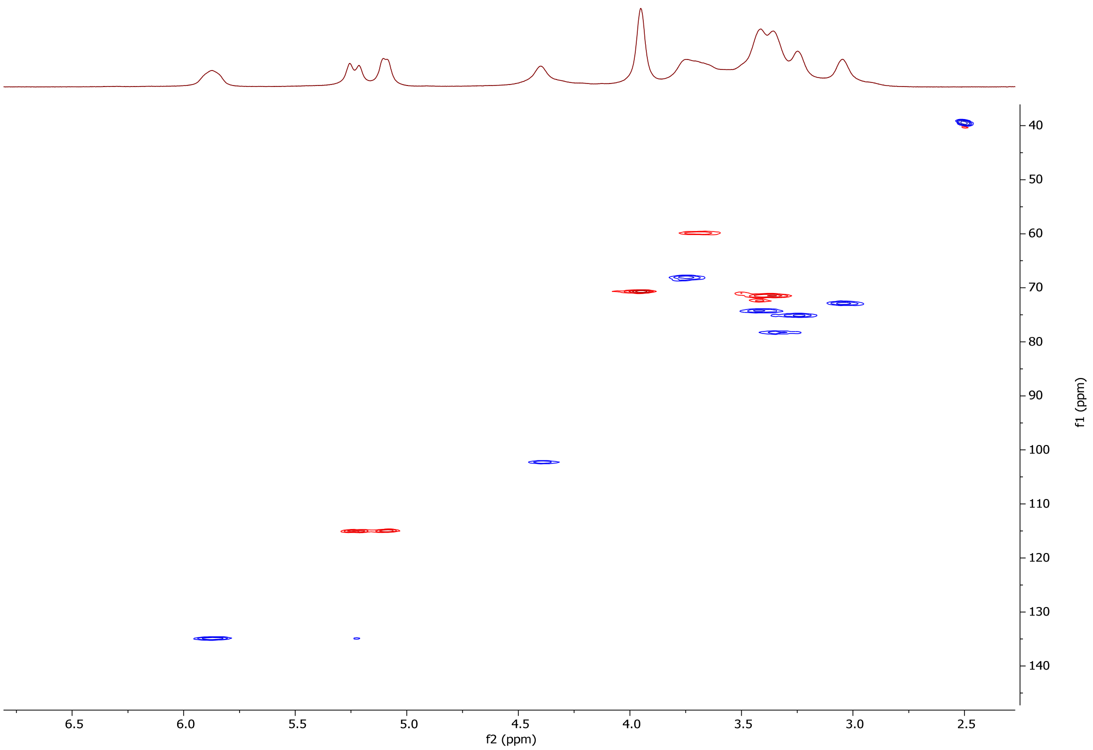

Figure S18. Multiplicity-edited HSQC spectrum ([P_{4444}][OAc]:DMSO-d_6 (1:4); 400 MHz ^{1}H frequency; 65°C) of the hDS-vCNC sample (5 wt%). CH_2 resonances are shown in red. CH / CH_3 signals are shown in blue. On top the diffusion edited ^{1}H spectrum is inserted. Only the zoom into the polysaccharide region is shown.

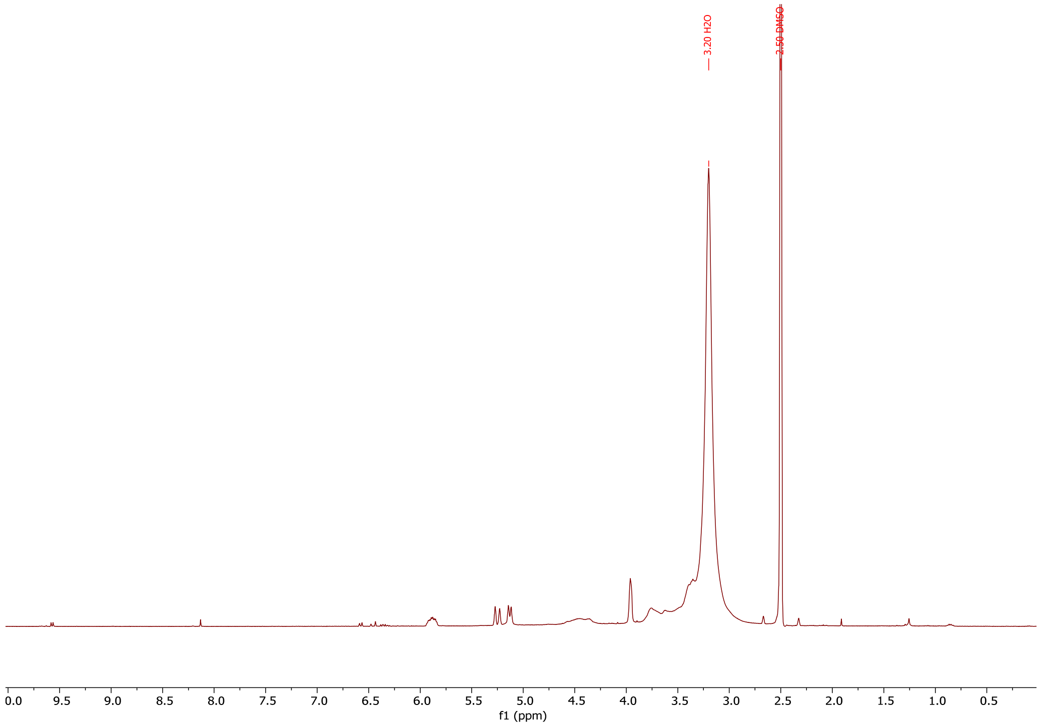

Figure S19. Quantitative ^{1}H NMR spectrum (DMSO-d_6; 400 MHz; 65°C) of the supernatant liquid obtained after stirring the hDS-vCNC sample (5 wt%) in DMSO-d_6 for 16 h. Only minimal amounts of allyl containing compounds and other unidentified molecules were extractable.

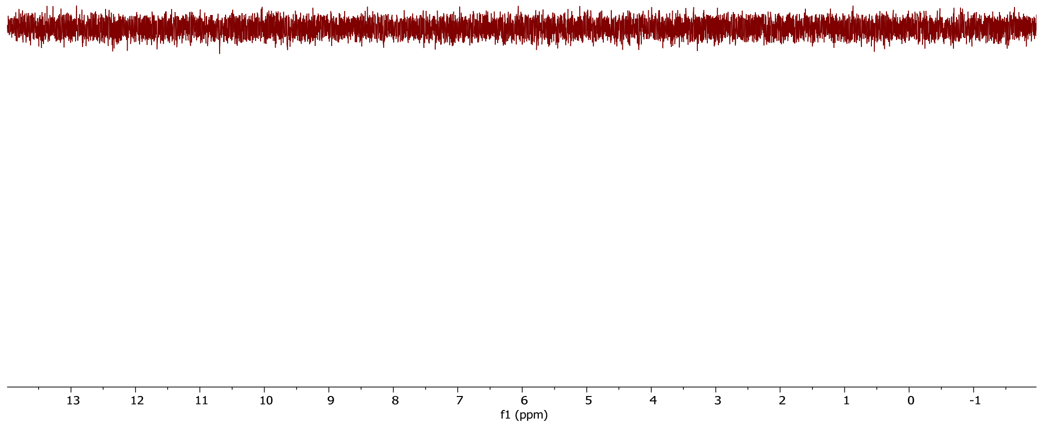

Figure S20. Diffusion edited ^{1}H NMR spectrum (DMSO-d_6; 400 MHz; 65°C) of the supernatant liquid obtained after stirring the hDS-vCNC sample (5 wt%) in DMSO-d_6 for 16 h. No peaks for dissolved macromolecules were visible.

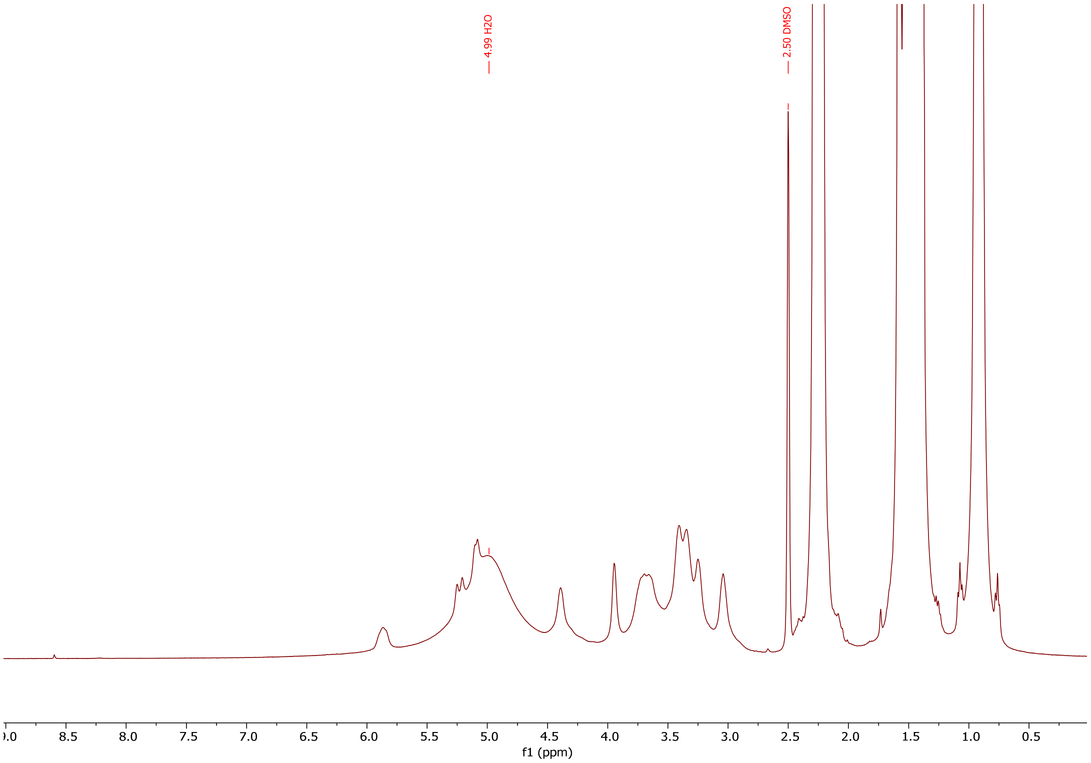

Figure S21. Quantitative ^{1}H NMR spectrum ([P_{4444}][OAc] : DMSO-d_6 (1:4); 400 MHz; 65°C) of the solid residue obtained after stirring the hDS-vCNC sample (5 wt%) in DMSO-d_6 for 16 h. Full spectral area with electrolyte and water resonances is shown.

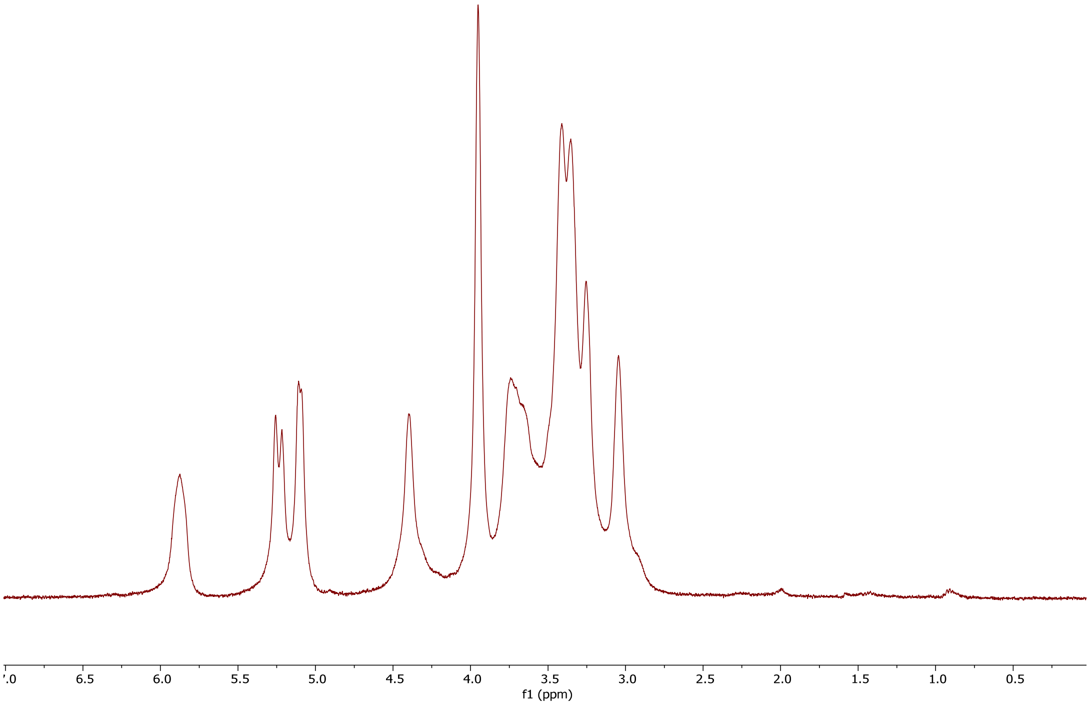

Figure S22. Diffusion edited ^{1}H NMR spectrum ([P_{4444}][OAc] : DMSO-d_6 (1:4); 400 MHz; 65°C) of the solid residue obtained after stirring the hDS-vCNC sample (5 wt%) in DMSO-d_6 for 16 h.

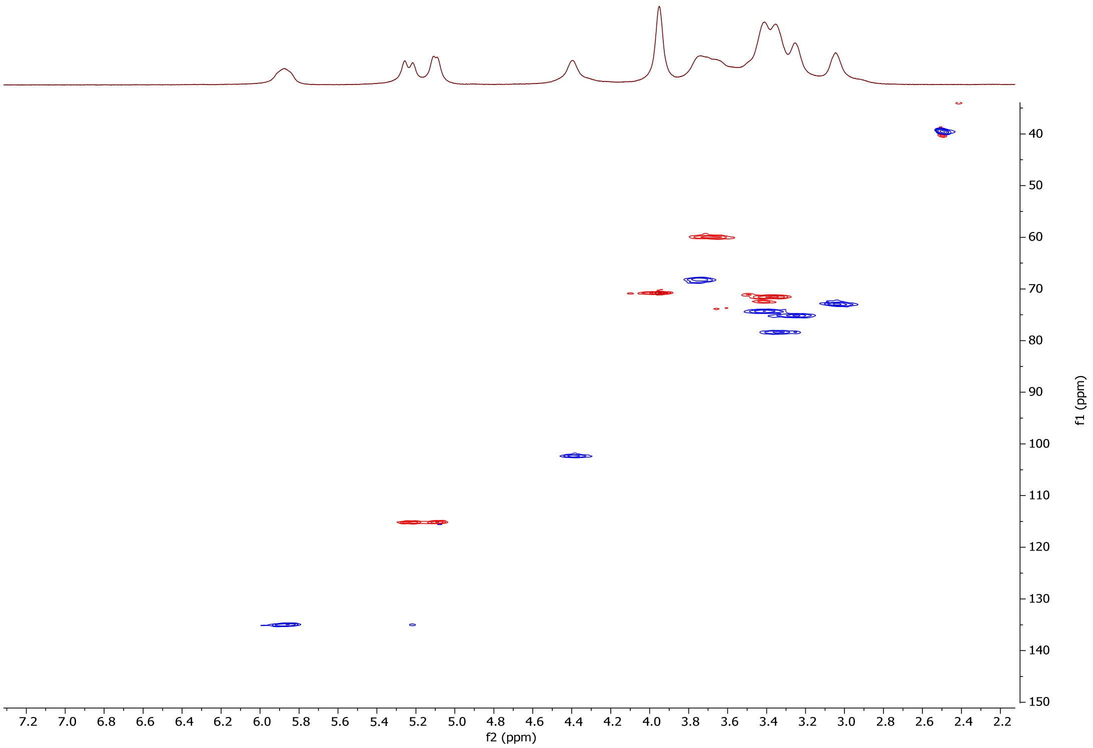

Figure S23. Multiplicity-edited HSQC spectrum ([P_{4444}][OAc]:DMSO-d_6 (1:4); 400 MHz ^1H frequency; 65°C) of the solid residue obtained after stirring the hDS-vCNC sample (5 wt%) in DMSO-d_6 for 16 h. CH_2 resonances are shown in red. CH / CH_3 signals are shown in blue. On top the diffusion edited ^1H spectrum is inserted. Only the zoom into the polysaccharide region is shown.

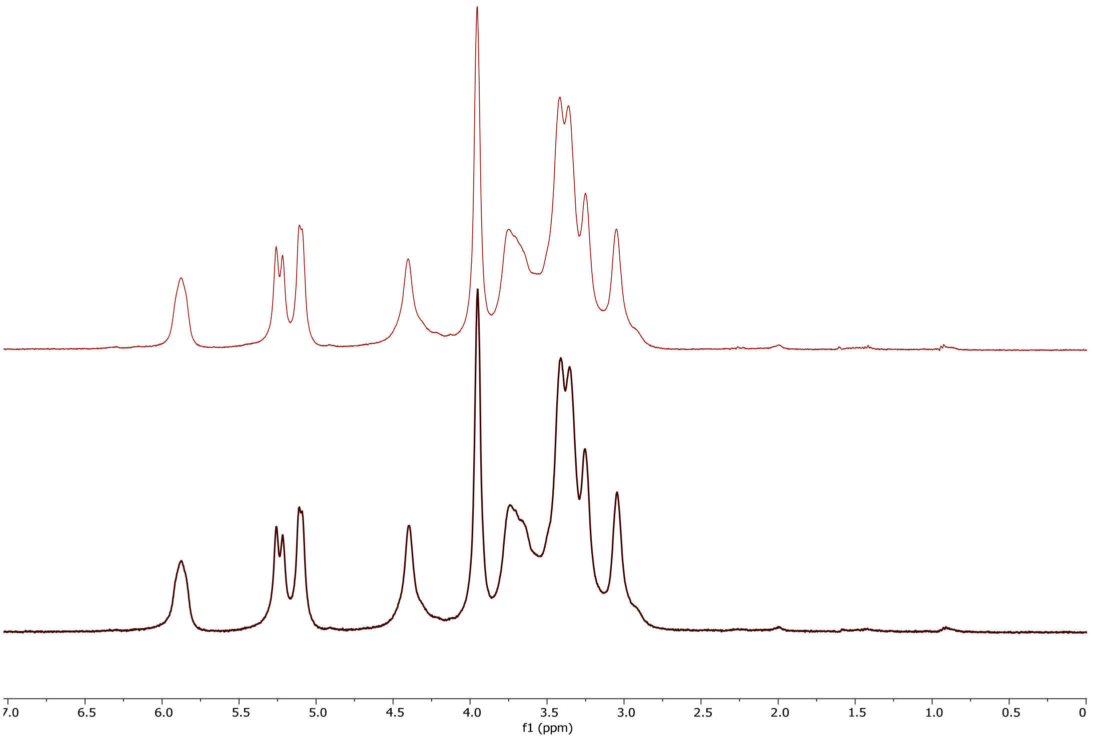

Figure S24. Comparison of the diffusion edited ^{1}H NMR spectra ([P_{4444}][OAc] : DMSO-d_6 (1:4); 400 MHz; 65°C) of the hDS-vCNC sample (5 wt%. **top**) and the solid residue of the hDS-vCNC sample after DMSO-d_6 extraction (5 wt%. **bottom**). The extraction did not result in significant changes in the spectra. suggesting a covalent modification of CNCs.

Peak fitting and calculation of vinyl content

To obtain approximate values for the amount of introduced vinyl moieties the quantitative 1H spectra of the lDS-vCNC and hDS-vCNC samples were evaluated using the peak fitting software *Fityk*. [3]

Due to peak broadening and superposition with the water signal aggressive baseline correction had to be applied (**Figures S25** and **S27**). The baseline corrected spectra were fitted using gaussian functions (**Figures S26** and **S28**). The confidence of the fit was evaluated by comparing the integrals of the C2-H and C1-H peaks of the glycopyranose unit and the C10-H_2, C11-H, C12-H_a and C12-H_b peaks, respectively (**Scheme S1**). A ratio of 1:1 can be expected for the C1-H and C2-H integrals. For the allyl functionality a ratio of 2:1:1:1 is expected. Except for the C12-H peaks the obtained values are in relatively good agreement (**Table S5**).

Table S5. Comparison of the peak areas obtained by fitting after baseline correction according to **Figures S25** and **S27**.

Entry	lDS-vCNC			hDS-vCNC		
	Area	Relative to C1-H	Relative to C10-H2	Area	Relative to C1-H	Relative to C10-H2
C1-H	10018	1,00	x	5617	1,00	
C2-H	10094	1,01	x	5473	0,97	
C10-H2	2508	x	2,00	4800	x	2,00
C11-H	1237	x	0,99	2572	x	1,07
C12-Ha	1152	x	0,92	2046	x	0,85
C12-Hb	1178	x	0,94	1918	x	0,80

It needs to be highlighted that different baseline settings were tried for the individual spectra and the obtained MS values for the lDS-vCNC sample varied between 0.10 to 0.13 and for the hDS-vCNC between 0.43 and 0.52.

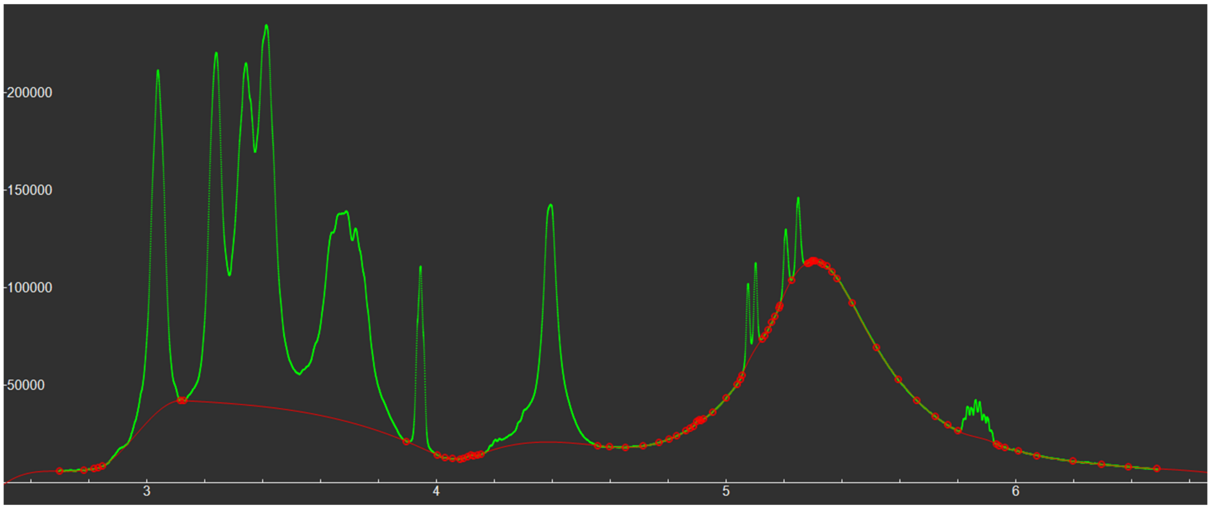

Figure S25. Baseline correction applied for the quantitative evaluation of the lDS-vCNC sample.

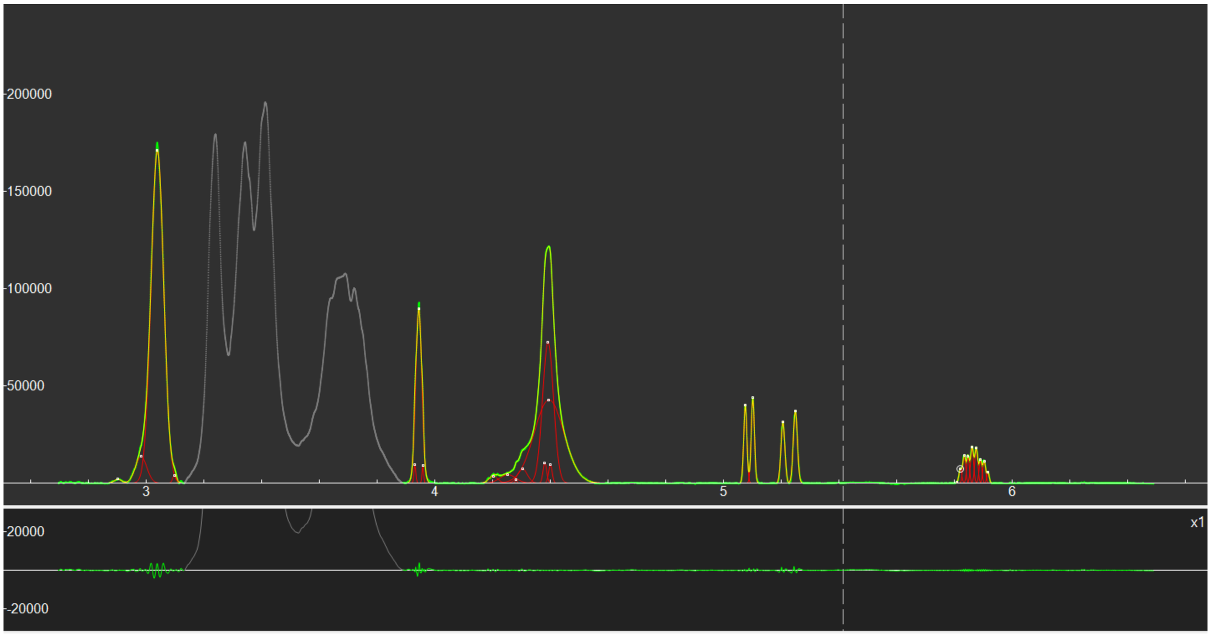

Figure S26. Gaussian deconvolution (**top**) and residual fitting error (**bottom**) for the lDS-vCNC sample.

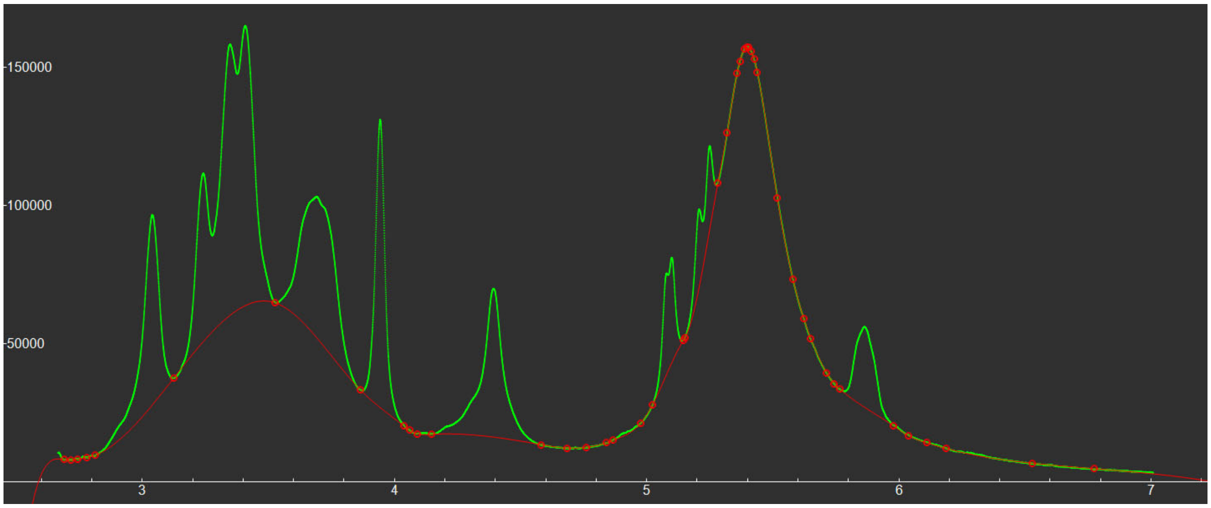

Figure S27. Baseline correction applied for the quantitative evaluation of the hDS-vCNC sample.

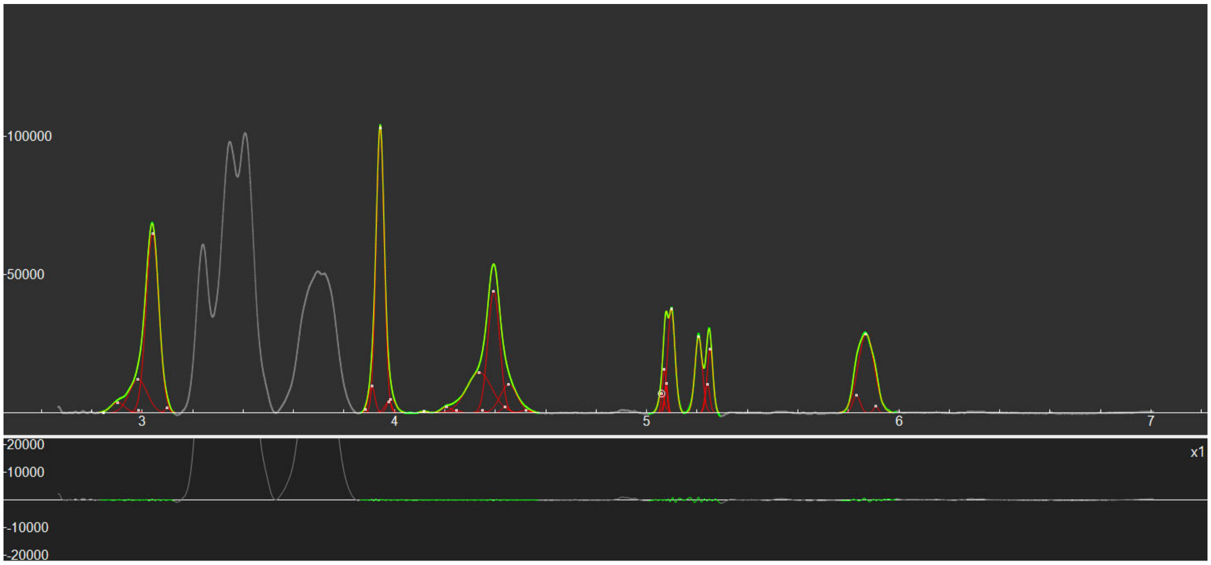

Figure S28. Gaussian deconvolution (**top**) and residual fitting error (**bottom**) for the hDS-vCNC sample.

References

[1] Qi, H.; Liebert, T.; Heinze, T. Homogenous synthesis of 3-allyloxy-2-hydroxypropyl-cellulose in NaOH/urea aqueous system. *Cellulose* **2012**, *19* (3), 925-932. DOI: 10.1007/s10570-012-9687-3.

[2] Pääkkönen, T.; Spiliopoulos, P.; Nonappa; Kontturi, K. S.; Penttilä, P.; Viljanen, M.; Svedström, K.; Kontturi, E. Sustainable High Yield Route to Cellulose Nanocrystals from Bacterial Cellulose. *ACS Sustain. Chem. Eng.* **2019**, *7* (17), 14384-14388. DOI: 10.1021/acssuschemeng.9b04005.

[3] Wojdyr, M. Fityk: a general-purpose peak fitting program. *J. Appl. Crystallogr.* **2010**, 43, 1126–1128. DOI: 10.1107/S0021889810030499.
